# Unlimited multistability and Boolean logic in microbial signalling

**DOI:** 10.1098/rsif.2015.0234

**Published:** 2015-07-06

**Authors:** Varun B. Kothamachu, Elisenda Feliu, Luca Cardelli, Orkun S. Soyer

**Affiliations:** 1 Systems Biology Program, College of Engineering, Computing and Mathematics, University of Exeter, Exeter, UK; 2 Department of Mathematical Sciences, University of Copenhagen, Universitetsparken 5, 2100 Copenhagen, Denmark; 3 Microsoft Research Cambridge, 7 JJ Thomson Avenue, Cambridge CB3 0FB, UK; 4 School of Life Sciences, University of Warwick, Coventry, UK

**Keywords:** multistability, prokaryotes, two component signalling networks, multi-domain proteins, Boolean logic, synthetic biology

## Abstract

The ability to map environmental signals onto distinct internal physiological states or programmes is critical for single-celled microbes. A crucial systems dynamics feature underpinning such ability is multistability. While unlimited multistability is known to arise from multi-site phosphorylation seen in the signalling networks of eukaryotic cells, a similarly universal mechanism has not been identified in microbial signalling systems. These systems are generally known as two-component systems comprising histidine kinase (HK) receptors and response regulator proteins engaging in phosphotransfer reactions. We develop a mathematical framework for analysing microbial systems with multi-domain HK receptors known as hybrid and unorthodox HKs. We show that these systems embed a simple core network that exhibits multistability, thereby unveiling a novel biochemical mechanism for multistability. We further prove that sharing of downstream components allows a system with *n* multi-domain hybrid HKs to attain 3*n* steady states. We find that such systems, when sensing distinct signals, can readily implement Boolean logic functions on these signals. Using two experimentally studied examples of two-component systems implementing hybrid HKs, we show that bistability and implementation of logic functions are possible under biologically feasible reaction rates. Furthermore, we show that all sequenced microbial genomes contain significant numbers of hybrid and unorthodox HKs, and some genomes have a larger fraction of these proteins compared with regular HKs. Microbial cells are thus theoretically unbounded in mapping distinct environmental signals onto distinct physiological states and perform complex computations on them. These findings facilitate the understanding of natural two-component systems and allow their engineering through synthetic biology.

## 1. Introduction

Cells are able to generate appropriate responses to diverse environmental stimuli. This ability requires mapping different environmental signals, or combinations thereof, onto specific physiological responses in a reliable fashion. Understanding the basis of this ability from the viewpoint of systems dynamics, as well as biochemical implementations, is thus crucial for the understanding of cellular behaviour in systems biology and its re-engineering in synthetic biology. At the level of system dynamics, multistable cellular systems such as signalling networks can display abrupt transitions among different steady states when changes in specific system parameters cross threshold points [1]. Furthermore, the threshold dynamics under multistabilty can allow cells to generate binary responses to environmental signals, thereby providing a potential to implement Boolean logic [2]. The threshold dynamics is the hallmark of multistability and is observed in several cellular responses including the all-or-none type responses seen in eukaryotic cell fate determination [3] and cell cycle regulation [4] and is indicated to underpin cellular differentiation [5].

From a mechanistic viewpoint, a key question is how multistability can be implemented through biochemical reactions. Answering this question could allow us to link observed biochemical features of natural systems to higher level response dynamics and exploit certain biochemistries for engineering cell behaviour. There has already been significant progress in both directions, with transcriptional feedback [5,6] and multi-site phosphorylation [7,8] identified as key biochemical mechanisms for implementing multistability. These mechanisms are found commonly in nature and have already been exploited in synthetic biology to engineer bistable gene expression and ultrasensitive signal processing [5,6,9–11]. In particular, multi-site phosphorylation is proposed as a very general mechanism to generate unbounded multistability [12,13]. It has been mathematically proven that a protein with *n* phosphorylation sites catalysed by enzymes in a distributive, sequential manner can give rise to at least *n* + 1 steady states [12,13]. Subsequent theoretical studies show that the sharing of enzymes (i.e. kinases and phosphatases) among the different phosphorylation steps and the linking of these steps are crucial prerequisites for multistability in a multi-site phosphorylation system [14,15].

Interestingly, multi-site, enzyme-mediated phosphorylation as seen in eukaryotic systems is mostly lacking in microbes. Instead, microbes rely on the so-called two-component systems for their environmental sensing and inter-cellular signalling [16]. Biochemically, two-component signalling is very distinct from enzyme-mediated phosphorylation dominating eukaryotic signalling and relies on phosphotransfer reactions between histidine and aspartate residues on histidine kinases (HKs) and response regulator (RR) proteins [16]. Since this biochemistry precludes the enzyme-mediated mechanisms of multistability generation described above, this raises the question of whether microbes use a different mechanism for generating multistability or lack this feature altogether. Although specific biochemical arrangements in some two-component systems are shown to enable bistability [17–19] and several microbial phenotypes are indicated to exhibit bistability [20,21], a general mathematical framework for assessing the capacity of system dynamics in two-component signalling has been lacking. Here, we develop such a framework and particularly consider the system dynamics arising from multi-domain HKs in two-component signalling. We find that the presence of these proteins can allow the system to display bistability, where systems with regular HKs cannot. We show that bistability arises from, and necessitates, the reactions among the different phosphorylation states of the multi-domain HK and a downstream protein. Extending from this result, we provide a mathematical proof to show that *n* multi-domain HKs sharing the same downstream component can result in a multistable system with 3*n* steady states. We find that this system dynamics property is easily used to implement Boolean logic using multi-domain HKs sensing different signals. Finally, we find that two experimentally studied systems, found in yeast osmoregulation and *Vibrio harveyi* quorum sensing, employ hybrid HKs and display a capacity to implement logic functions and bistability with hysteresis as expected by the presented theoretical framework.

## 2. Results

**Figure 1.**
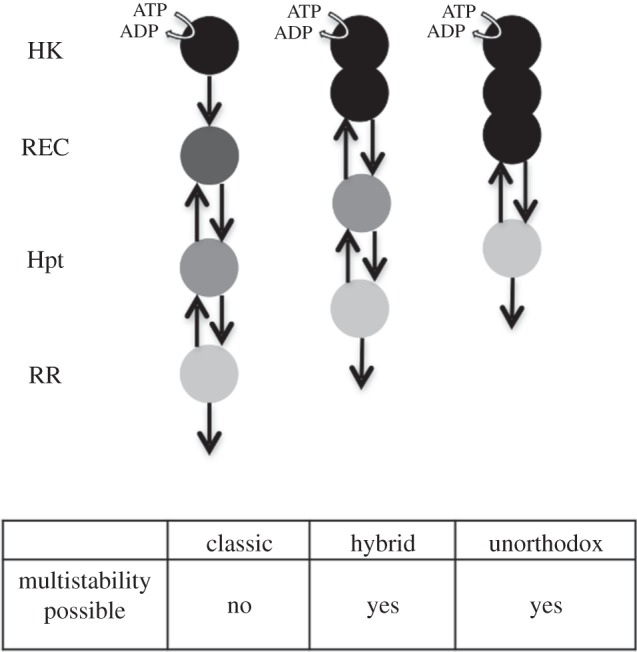
Cartoon representation of phosphorelays embedding the different types of biologically observed HKs and their ability to attain multistability. Multistability was first assessed using the chemical reaction network toolbox v. 2.2 (http://www.crnt.osu.edu/CRNTWin/) and further analysed by solving the steady state equations (see the electronic supplementary material, SI-1).

Two-component signalling systems comprising HKs and cognate RRs [16] are found in all studied microbial genomes to date, with some environmental bacteria shown to contain more than 60 distinct two-component systems [22,23]. The response dynamics in a few of these systems, most notably those regulating the chemotaxis and sporulation responses, are characterized in detail [24,25]. Here, we focus on developing a general mathematical framework to capture and analyse the system dynamics emerging from two-component signalling. At its core, two-component signalling comprises a cognate HK–RR pair. Upon receiving a signal, the HK can auto-phosphorylate on a histidine residue, and subsequently transfer the phosphate group to an aspartate residue on the RR [16]. In the case of a single HK–RR pair, there is only one phosphotransfer reaction between the two proteins; while in the case of so-called phosphorelays, there are usually three distinct phosphotransfer reactions [26]. These reactions involve the HK and the RR at the beginning and end of the relay, respectively, and the two intermediate proteins containing so-called receiver (REC) and histidine-phosphotransfer (Hpt) domains [26]. These four stages of the phosphorelay can be encoded on separate proteins as seen for example in the phosphorelay regulating *Bacillus subtilis* sporulation decision, or the REC and Hpt domains can be embedded into a single protein known as hybrid HK (embedding REC domain only) or unorthodox HK (embedding both REC and Hpt domains) [27] (figure 1). All three types of HKs, regular, hybrid and unorthodox, are found to coexist in many microbial genomes, as well as in plants [27–29]. Having multiple domains, the hybrid and unorthodox HKs are similar to eukaryotic signalling proteins with multiple phosphorylation sites, raising the question of whether these HKs have a functional significance in terms of systems' steady-state behaviour and information processing capacity.

### 2.1. Multi-domain histidine kinases enable multistability

To address this question, we created mathematical models of a phosphorelay embedding a regular, hybrid or unorthodox HK (see Material and methods and electronic supplementary material, SI-1). All of these models implemented forward and reverse phosphotransfer among relay components and hydrolysis reactions at the levels of REC and RR. These reactions are shown to occur in experimental studies [30,31] and were included in previous theoretical models of phosphorelays [24,32,33]. The analysis of the resulting chemical reaction systems for the three models revealed that the system with the regular HK does not fulfil the theoretical requirements for bistability, as shown before [33], while the systems with hybrid and unorthodox HKs do (see figure 1 and electronic supplementary material, SI-1).

**Figure 2.**
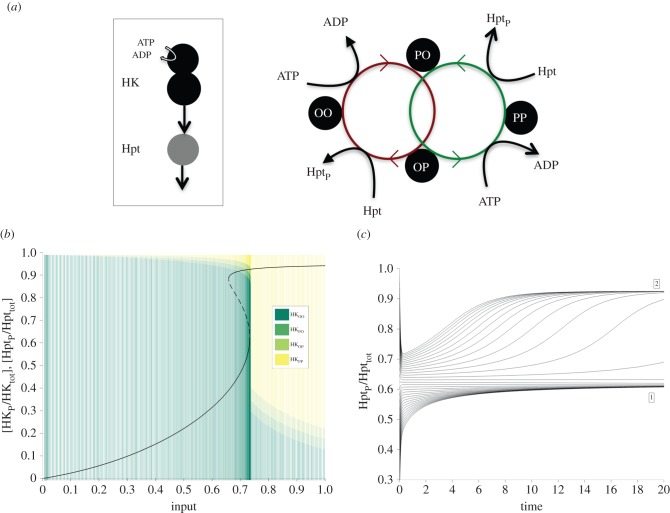
(*a*) Cartoon representation of the minimal core system using a hybrid HK and that maintains the ability for multistability. The right-hand cartoon shows the two connected feedback loops inherent in this system. The four phosphorylation states of the two-domain hybrid HK are shown using the labels O (unphosphorylated site) and P (phosphorylated). Auto-phosphorylation of the first site (i.e. states OO and OP) involves conversion of ATP to ADP, while de-phosphorylation of the second site (i.e. states OP and PP) involves conversion of Hpt to Hpt_P_ as shown. (*b*) Bifurcation plot (i.e. dose–response curve) for the minimal core system, for a specific set of parameters (see the electronic supplementary material, table S1). The plot shows the fraction of phosphorylated Hpt at steady state for a given input level (the change in input level is simulated by varying the auto-phosphorylation rate constants of HK, *k*_1_ and *k*_3_, while keeping the ratio *k*_3_/*k*_1_ fixed). The solid and dotted lines indicate stable and unstable steady states, respectively. The fractions of the different phosphorylation states of HK for the steady states are shown as a stacked bar (overlay). The PP state is populated only above a threshold input level as discussed in the main text. (*c*) Time-series plot for the system shown in (*b*). The phosphorylated Hpt levels approach the two different stable steady states depending on the initial conditions.

To better understand the source of bistability in the multi-domain HKs, we have focused on the hybrid HK. There, the HK protein can be modelled as an entity with four states, which can be denoted as OO, PO, OP and PP, corresponding to the different phosphorylation status of the HK and REC domains (figure 2). We simplify this model by systematically removing reactions and species from it, to obtain a minimal system that still maintains ability for bistability. This minimal, *core* system did not require the presence of RR, reverse phosphorylation reaction from the Hpt to the hybrid HK, nor the hydrolysis reaction from the REC domain (figure 2). We analytically solved the steady-state equations arising from the set of ordinary differential equations describing the dynamics of this core system (see electronic supplementary material, SI-1). This allowed us to derive a set of necessary and sufficient conditions on the reaction rate constants and total concentrations that endow the system with bistability (see electronic supplementary material, SI-1).

### 2.2. Reactions among histidine kinase domains and the downstream target give rise to interconnected feedback loops

While the mathematical complexity of these conditions does not permit a simple biological interpretation, one notable necessary condition for bistability in this core model is that the rate constant of auto-phosphorylation of the hybrid HK must be higher when the REC site is phosphorylated compared with when it is not (i.e. the auto-phosphorylation of the OP state must be higher than that of the OO state, *k*_3_ > *k*_1_). Interestingly, these two reactions drive two connected feedback loops, where one loop cycles from the OO state, to PO, OP and then back to OO, while the other cycles from the PO state, to OP, PP and then back to PO (figure 2). This observation allows an intuitive understanding of bistability in this core system. At low-signal and high-Hpt levels, the auto-phosphorylation of the HK can be balanced between a flow of phosphate groups through Hpt, allowing the first loop (OO–PO–OP–OO) to dominate the dynamics. As the signal increases and the Hpt is consumed more and more, this balance is increasingly disrupted and there is suddenly not enough Hpt to absorb all of the phosphate groups from the OP state. This then allows the OP to increasingly undergo auto-phosphorylation, which happens faster under the condition of *k*_3_ > *k*_1_, and leads to the second loop (PO–OP–PP–PO) to start dominating. This results in a sudden rise in PP and the phosphotransfer rate to Hpt, overwhelming the latter and causing its phosphorylated state to make a sudden jump. This jump is the bifurcation point that we observe in the system dynamics. We find that this intuitive narrative fits with the observed temporal and steady-state concentrations of the different phospho-states of the HK (figure 2) and also explains the effect of increasing the ratio *k*_3_/*k*_1_ on the system dynamics (electronic supplementary material, figure S1).

The aforementioned two feedback loops are complemented by a third feedback loop that becomes visible when we display the core model as a bipartite reaction graph (electronic supplementary material, figure S2). It is known that bistability requires at least one positive feedback loop in such a graph [34,35]; however, we find that alternative reaction schemes of the same size as the core system and implementing one or more feedback loops do not allow for bistability (electronic supplementary material, figure S2). This shows that the reaction scheme in the core of the hybrid HK structure implements a particular, non-trivial mechanism for generating bistability. This mechanism is still intact in the full hybrid and unorthodox HK models, nested within a more complex reaction scheme that includes hydrolysis and reverse-phosphotransfer reactions. We find that these additional reactions allow tuning the exact shape of the input–output response dynamics, with reverse phosphorylation providing the possibility of achieving more pronounced switch-like dynamics (electronic supplementary material, figure S3). More broadly, we show that the mathematical findings for multistability extend to the full hybrid and unorthodox HK models, even when we take into account complex formation in the phosphotransfer reactions (see electronic supplementary material, SI-1).

### 2.3. Sharing of downstream target among multi-domain histidine kinases leads to unbounded multistability and implementation of Boolean logic

**Figure 3.**
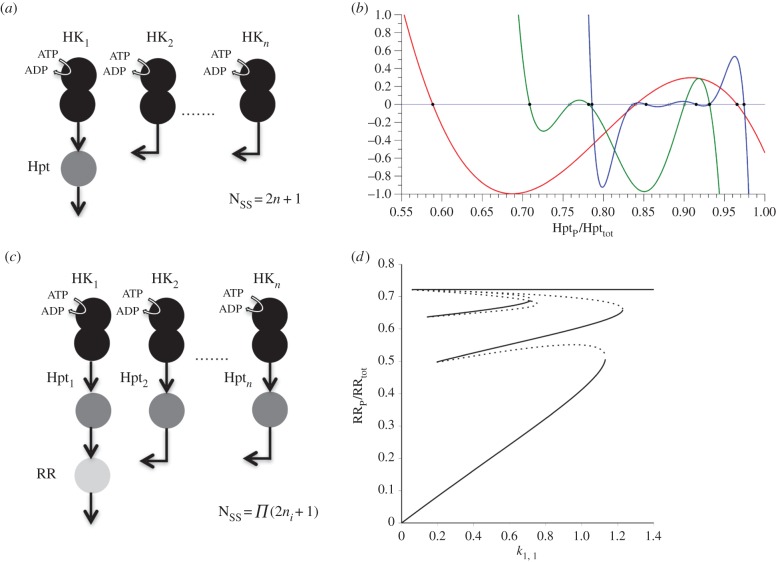
Cartoon representation of sharing of downstream components at the level of Hpt (*a*) or RR (*c*). (*b*) Plot of the polynomial function (see the electronic supplementary material, SI-1) characterizing the steady-state level of the phosphorylated Hpt for the system shown in (*a*) for a specific set of parameter values (see the electronic supplementary material, table S1). Both the polynomial and the variable are scaled to the shown window. The polynomial is plotted in red, green and blue for one, two and three HKs, respectively. Each crossing of the polynomial with the *x*-axis is a steady state of phosphorylated Hpt. Stable and unstable steady states are shown as filled and open circles, respectively. (*d*) Bifurcation plot for a system with two hybrid HKs, each with separate Hpts that share the same RR (as shown in (*c*), for *n* = 2). We assume that the auto-phosphorylation rate constants for HK_1_ and HK_2_ (when the REC site is unphosphorylated) are the same and determined by the same signal (i.e. *k*_1,1_ = *k*_2,1_). The bifurcation plot shows the fraction of phosphorylated RR at steady state for a given input level (*k*_1,1_ = *k*_2,1_). The parameter values are chosen such that the system displays nine distinct steady states (see the electronic supplementary material, table S1). The solid and dotted lines indicate stable and unstable steady states, respectively.

The key mechanisms for generating bistability in a single multi-domain HK are the feedback loops among its internal phospho-states and the interlinkage of these to a downstream target. This raises the possibility that component sharing, in which several multi-domain HKs share (i.e. phosphotransfer to) the same downstream target can lead to an increase in the number of steady states in the system. To address this possibility, we analysed a generalized model of *n* HKs that transfer a phosphate group to a common Hpt. We prove mathematically that such a system can attain 2*n* + 1 steady states under appropriate choices of parameters; to this end, we show that the steady states of a system comprising *n* HKs that transfer a phosphate group to the same Hpt are in correspondence with the positive roots of a polynomial of degree 2*n* + 1 in the concentration of phosphorylated Hpt (electronic supplementary material, SI-1; and figure 3). Of these steady states, *n* are proved to be unstable, and simulations show that the remaining *n* + 1 steady states are, as expected, stable. Considering component sharing at the level of RR, we show that the system with *m* modules that phosphotransfer to the same RR, and where the *i*th module comprises *n_i_* hybrid HKs sharing a single Hpt, allows for *Π*(2*n_i_* + 1) steady states (where the multiplication is over the *m* modules; figure 3*d*). In particular, the system comprising *n* phosphorelays, each consisting of a hybrid HK and an Hpt domain that transfers a phosphate group to a common RR, can attain 3*n* steady states.

**Figure 4.**
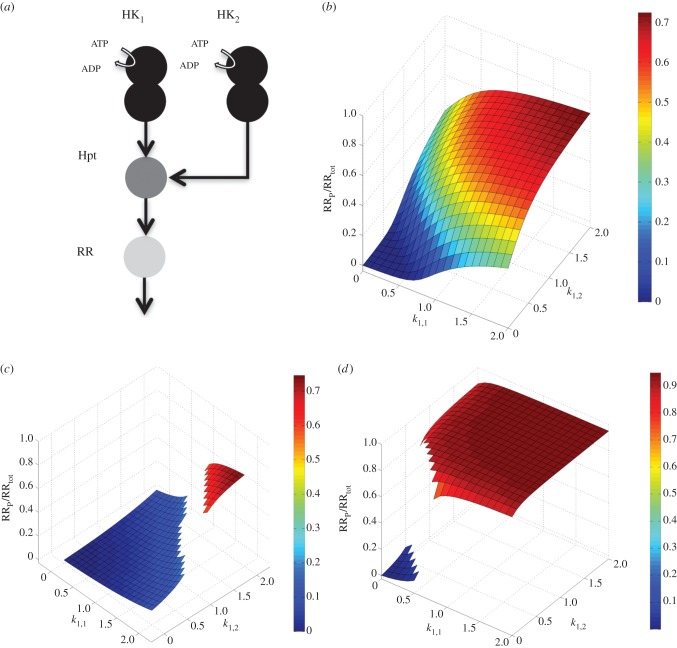
(*a*) Cartoon representation of a system with two hybrid HKs that share the same Hpt. (*b*–*d*) Implementation of different signal processing functions using the system shown in (*a*); an ‘adder’ as seen experimentally in *V. harveyi* [36] (*b*), a Boolean AND gate (*c*) and a Boolean OR gate (*d*). Each panel shows the fraction of phosphorylated RR at steady state for different auto-phosphorylation rate constants (i.e. input levels) acting on the two HKs. It is assumed that the two signals are specific for the two HKs (*k*_1,1_, *k*_1,3_ and *k*_2,1_, *k*_2,3_, respectively) and the ratios *k*_1,1_/*k*_1,3_ and *k*_2,1_/*k*_2,3_ are held fixed. The systems shown in (*c,d*) are bistable, with the blank regions of the surfaces corresponding to unstable regions. For the parameters used, see the electronic supplementary material, table S1.

These mathematical proofs show that microbes can use individual hybrid and unorthodox HKs to implement multistability and are theoretically unbounded in their capacity to expand the number of available steady states through sharing of downstream components (Hpt or RR) among such HKs. We find that component sharing among multi-domain HKs can also be used flexibly, and in other ways. For example, component sharing at the level of RR and using HKs sensing the same signal can be used to implement *n* bistable switches with distinct threshold signal levels (electronic supplementary material, figure S4). Perhaps more interestingly, HKs sensing different signals and component sharing at the level of RR can be used to implement Boolean logic gates. In particular, we could identify a simple architecture involving two HKs, sharing the same Hpt, that can implement an AND and OR gate (figure 4). The system could be tuned between implementing these different logic gates simply by changing the total concentrations of components and the dephosphorylation rate of phosphorylated RR (see the electronic supplementary material, table S1). Furthermore, based on the above mechanistic understanding and parameter sampling, we could identify parameter combinations for the same system that allowed summation over the two signals (figure 4*b*).

### 2.4. Multi-domain histidine kinases are common in microbial genomes and known systems embedding them display bistability under biologically feasible parameters

Hybrid (and unorthodox) HKs occur commonly in microbial genomes, and experimentally studied model systems display dynamics as expected from the theoretical analysis. The theoretical findings presented so far suggest that microbes could use hybrid and unorthodox HKs to implement multistability, threshold dynamics and Boolean logic in two-component systems. To study to what extent these proteins are prevalent in the microbial world, we used an existing database dedicated to two-component signalling [37,38]. We found that regular, hybrid and unorthodox HKs coexist in all microbial genomes with annotated two-component systems, although different genomes contain different proportions of these proteins (electronic supplementary material, figure S5). While a simple analysis of the ratio of multi-domain (i.e. hybrid and unorthodox) to regular HKs against genome size did not result in any significant pattern (electronic supplementary material, figure S5), we hypothesize that this ratio may correlate with some environmental or ecological features.

**Figure 5.**
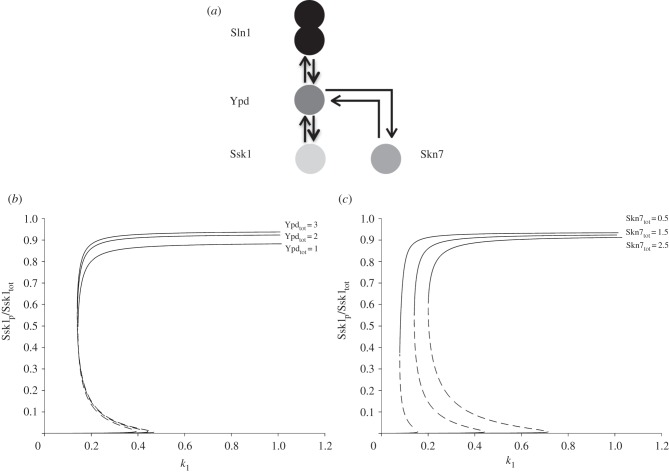
(*a*) Cartoon representation of the yeast two-component system involved in osmosensing. This system contains a hybrid HK (Sln1), that transfers a phosphate group to an Hpt (Ypd), which subsequently transfers a phosphate group to two RRs (Ssk1 and Skn7). (*b,c*) Bifurcation plots for the system shown in (*a*), for a specific set of parameters combining the experimentally measured rates in [30] with biologically feasible parameter values (see the electronic supplementary material, table S2 for parameters and electronic supplementary material, SI-2 for the computer-executable full model of this system). The bifurcation plot shows the fraction of phosphorylated Ssk1 at steady state for a given input level. The change in input level shown on the *x*-axis is simulated by varying the auto-phosphorylation rate constants of Sln1, *k*_1_ and *k*_3_, while keeping the ratio *k*_3_/*k*_1_ fixed. (*b*) Shows the effect of increasing the total concentration of Ypd (from 1, to 2, to 3), while (*c*) shows the effect of increasing the total concentration of Skn7 (from 0.5, to 1.5, to 2.5) on the bifurcation plot. In each plot, the solid and dashed lines correspond to stable versus unstable steady states.

Despite the high prevalence of hybrid and unorthodox HKs, detailed experimental studies of these systems are rare. The most well-studied cases are those involved in the quorum sensing system of *Vibrio harveyi* [36] and the osmosensing system of yeast [30]. The former system implements three hybrid HKs that share the same Hpt, and, as such, closely resembles one of the systems considered in this work. Experiments with a modified version of this quorum sensing system involving just two HKs have shown that the ability to perform a summation as shown in figure 4 is possible in a natural system [36]. In the yeast osmosensing system, a hybrid HK transfers a phosphate group to two downstream RRs as schematically shown in figure 5*a*. While we do not have experimental data on response dynamics of this system, *in vitro* phosphotransfer experiments provide kinetic rate measurements for some of the phosphotransfer reactions [30]. We have developed a model of this system, which is provided as an executable model in the electronic supplementary material, SI-2. In this model, we considered the experimentally measured kinetic rates and set the remaining parameters in a biologically feasible regime (see the electronic supplementary material, table S2). The analysis of this model shows that this system exhibits bistability. Furthermore, we observe a significant level of hysteresis, that is, the threshold point for switching between the two stable steady states depends highly on whether the signal is being increased or decreased (figure 5*b*).

## 3. Discussion

The ability to map environmental signals onto distinct internal physiological states or programmes is expected to be critical for single-celled microbes that often need to respond to signals arising from fluctuating environments and neighbouring populations. This physiological capacity usually requires signalling systems that can implement threshold dynamics or multistability. While previous studies have identified multi-site phosphorylation as a key biochemical mechanism to attain unbounded multistability, this mechanism is mostly lacking from microbial cells. Instead these cells rely on phosphotransfer reactions in two-component signalling for their environmental information processing. Here, we show that hybrid HKs can enable bistability through embedding multiple feedback loops within their own reaction scheme. Furthermore, we show that the presence of this feature is preserved in systems embedding the more complex unorthodox HKs or architectures with multiple hybrid HKs.

When multiple copies of hybrid HKs are sharing the same downstream component, we find that the system can attain unbounded multistability. In particular, we derive several mathematical proofs relating the number of multi-domain HKs sharing the same component and the number of steady states available to the system. These proofs extend to models considering complex formation and show that microbes can attain unbounded multistability by employing two-component signalling. Furthermore, we find that the same principle of component sharing among multi-domain HKs can be used to implement Boolean logic gates when different HKs sense different signals.

The presented theoretical framework fits well with the few experimentally studied cases involving hybrid HKs. The quorum sensing system of *Vibrio harveyi* implements component sharing as discussed above. It has been experimentally shown that the system implements an ‘adder’ function [36] that could be readily reproduced with the models presented here. Similarly, we found that the osmosensing system from yeast, implementing a hybrid HK [30], displays bistability and hysteresis under an experimentally measured and biologically feasible parameter regime. These analyses lend further support to the idea that the observed capacity for multistability arising from multi-domain HKs is exploited by evolution and is implemented in natural two-component systems.

We argue that component sharing among multi-domain HKs could be seen as a ‘design principle’, which microbial cells can use flexibly to generate unbounded numbers of physiological steady states and to implement logic operations. While systematic analyses in *Escherichia coli* and *Caulobacter crescentus* found mostly distinct HK–RR pairs [39,40], and a recent study suggested rapid diversification of RRs after duplication [41], these studies focused primarily on regular HKs. Where analysed, specific two-component systems involving multi-domain HKs are found to display significant cross-talk [42] and also the exact type of component sharing described here (as seen in *Vibrio harveyi* [36]). Furthermore, all analysed microbial genomes that display two-component systems feature regular, hybrid and unorthodox HKs, and in some cases the ratio of the multi-domain HKs to the regular HKs is well above 1 (see the electronic supplementary material, figure S5).

The theoretical findings presented here also point to hybrid and unorthodox HKs as ideal targets for engineering artificial and controllable multistable systems. This would extend the repertoire of synthetic biology, where engineering of two-component systems has so far only concentrated on exploiting their signal sensing properties [43,44] rather than signal processing capacities. In particular, the capacity for these systems to implement multiple bistable switches that can be controlled at different signal levels or through a combination of signals can allow construction of synthetic logic gates at the level of signalling pathways.

## 4. Material and methods

We develop generic models of four layered phosphorelays embedding a regular, hybrid or unorthodox HK (figure 1). These models incorporate experimentally observed reverse-phosphorylation reactions between the REC-Hpt and Hpt-RR proteins (domains), and hydrolysis reactions from REC and RR [24,30–33]. The hydrolysis reactions are considered possible only on REC and RR, as these proteins are phosphorylated on an aspartate residue (while HK and Hpt are phosphorylated on a histidine residue), which has an inherent instability when phosphorylated [45]. We model inter-domain phosphotransfer as bi-molecular reactions, while transitions among the internal states in the multi-domain HKs [46] as first-order reactions (see electronic supplementary material, SI-1).

### 4.1. Full, core and multi-histidine kinase reaction systems

Using the above general considerations, we wrote reactions for the different systems and derived the corresponding ordinary differential equations (see electronic supplementary material, SI-1). The reaction network for the full system with a hybrid HK is4.1
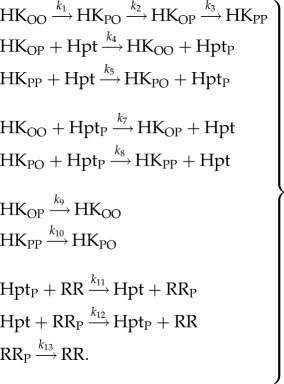
Here the HK protein is modelled as an entity with four states, which are denoted as OO, PO, OP and PP, corresponding to the different phosphorylation status of the HK and REC domains. The reaction network for the full system with an unorthodox HK is implemented similarly, but modelling the HK as an entity with eight states (see electronic supplementary material, SI-1 §4.3). The network shown in equation (4.1) is bistable. We determine a core system, which still maintains bistability, by removing reactions of the full reaction system with a hybrid HK until bistability is lost. This results in a core system that implements the reactions corresponding to the reaction rate constants *k*_1_ … *k*_5_ (reactions at the top in equation (4.1)), together with a hydrolysis reaction from Hpt_P_:4.2
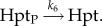
The systems with multiple HKs are developed using the core or full reaction systems and by repetition of specific reactions for new HKs and possibly new Hpt, and where either Hpt or RR is shared (see electronic supplementary material, SI-1 §§2 and 3).

### 4.2. Analysis of the steady states of the reaction systems

Under the assumption of mass action kinetics, the evolution of the concentrations in time of each of the reaction system is modelled with a system of ordinary differential equations (see electronic supplementary material, SI-1). We first undertake a detailed mathematical analysis of the steady-state equations of the core system (electronic supplementary material, SI-1 §1). We show that the positive steady states are in one-to-one correspondence with the positive roots of the following polynomial in the concentration of Hpt (see electronic supplementary material, SI-1 equation (S9)):4.3
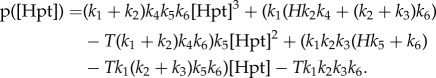
Here the reaction rate constants are as shown in equations (4.1) and (4.2) and the parameters *T* and *H* stand for the total concentrations of Hpt and hybrid HK, respectively. Using that *T* = [Hpt] + [Hpt_P_], the polynomial is easily transformed into a polynomial in the concentration of phosphorylated Hpt. Since the degree of the polynomial is 3, this polynomial has at most three positive roots. We show in the electronic supplementary material, SI-1 that there exist choices of rate constants and total concentrations such that the system has indeed three positive steady states, i.e. displays bistability (figure 2). Furthermore, we derive specific necessary and sufficient conditions on the parameters for the presence of bistability (see electronic supplementary material, SI-1 §1.3).

We generalize the above analysis to systems comprising *n* hybrid HKs sharing the same downstream Hpt. In this case, we show that the positive steady states are in one-to-one correspondence with the positive roots of a polynomial of degree 2*n* + 1 in the concentration of Hpt*,* implying that there can at most be 2*n* + 1 positive steady states. The coefficients of the polynomial depend on the reaction rate constants and the total concentrations of each HK and Hpt. We subsequently show that there is always a choice of parameters such that the 2*n* + 1 roots of the polynomial are all positive, thereby giving a choice of parameters such that the system has 2*n* + 1 positive steady states (see electronic supplementary material, SI-1 §2).

The results obtained for the core system are used to determine how many steady states the full systems can have. We consider several combinations in which Hpt is shared and in which RR is shared. Our reasoning involves the use of general results on chemical reaction networks modelled with mass action kinetics. Specifically, we use that if a network admits *N* steady states, then the network obtained after making some reactions reversible [47] or adding intermediate complex formation [48] can also admit at least *N* steady states. Further, the local stability properties of the steady states are preserved (see the electronic supplementary material, SI-1).

### 4.3. Analysis of response dynamics of reaction systems

To determine whether reaction systems with multiple hybrid HKs can implement response dynamics mimicking Boolean logic, we developed models where different HKs respond to different signals. We ran temporal dynamics with selected reaction rate constants and total concentrations. In particular, we used the mechanistic understanding for the generation of bistability in the system (see main text) to choose parameters that are expected to implement specific response dynamics, including Boolean logic. Representative parameter sets are found and simulated using different signal levels. For each combination of signal level, the systems were run to steady state. The system was deemed at a stable steady state when changes in the system's output variable (i.e. phosphorylated forms of the proteins) were lower than 10^–5^. All simulations were run using Matlab and its native ODE solvers (ODE15s). (Simulation scripts are available upon request.)

## Supplementary Material

kothamachu_etal_supplementary_information_SI-1

## Supplementary Material


