# Supplementary material for: Unlimited multistability and Boolean logic in microbial signalling
Source: J R Soc Interface. 2015 Jul 6;12(108):20150234. doi: 10.1098/rsif.2015.0234 (PMC4528588; doi:10.1098/rsif.2015.0234)
Supplement: kothamachu_etal_supplementary_information_SI-1 [file rsif20150234supp1.pdf]

Unlimited multistability and Boolean logic in microbial signaling

Supplementary Text
Proof of mathematical statements

Varun B Kothamachu, Elisenda Feliu, Luca Cardelli, and Orkun S Soyer

May 27, 2014

Contents

1 The core model for the hybrid histidine kinase **1**
1.1 Model description . . . 1
1.2 Positive steady states . . . 2
1.3 Necessary conditions for bistability . . . 4
1.4 Necessary and sufficient conditions for bistability . . . 4

2 The core model for n hybrid HKs competing for the same Hpt **5**
2.1 Model description . . . 5
2.2 Positive steady states . . . 6
2.3 Existence $2n+1$ positive steady states. . . . 7
2.4 n unstable steady states . . . 10

3 Multiple phosphorelays with a common RR **11**
3.1 The core model for M hybrid phosphorelays competing for an RR . . . 11
3.2 Multiple positive steady states . . . 12

4 From core to full models **15**
4.1 Theoretical results . . . 15
4.2 Full hybrid HK . . . 15
4.3 Unorthodox HK . . . 16

5 Proof of the lemmas **18**
5.1 Proof of Lemma 1 . . . 18
5.2 Proof of Lemma 2 . . . 18
5.3 Proof of Lemma 3 . . . 19
5.4 Proof of Lemma 4 . . . 20

1 The core model for the hybrid histidine kinase

1.1 Model description

We consider a core model for a two component system with hybrid histidine kinase (hybrid HK) and one Hpt. The hybrid HK consists of two phosphorylable domains. We denote the phosphorylation state of each site by P if the site is phosphorylated and 0 if it is not, such that the four possible states of HK are denoted by HK_{00}, HK_{P0}, HK_{0P}, and HK_{PP}. We let Hpt be

the unphosphorylated His-containing phosphotransfer protein, and $\mathrm{Hpt_P}$ the phosphorylated form.

The reactions we consider are the following:

$$\mathrm{HK_{00}} \xrightarrow{k_1} \mathrm{HK_{P0}} \xrightarrow{k_2} \mathrm{HK_{0P}} \xrightarrow{k_3} \mathrm{HK_{PP}} \qquad \mathrm{HK_{0P}} + \mathrm{Hpt} \xrightarrow{k_4} \mathrm{HK_{00}} + \mathrm{Hpt_P}$$

$$\mathrm{Hpt_P} \xrightarrow{k_6} \mathrm{Hpt} \qquad \mathrm{HK_{PP}} + \mathrm{Hpt} \xrightarrow{k_5} \mathrm{HK_{P0}} + \mathrm{Hpt_P}.$$

For notational convenience, we name the rate constants $k_1, \ldots, k_6$, which differs from the notation in the main text. We denote the concentration of the species as follows:

$$\begin{array}{llll} x_1 := [\mathrm{HK_{00}}] & x_2 := [\mathrm{HK_{P0}}] & x_3 := [\mathrm{HK_{0P}}] & x_4 := [\mathrm{HK_{PP}}] \\ x_5 := [\mathrm{Hpt}] & x_6 := [\mathrm{Hpt_P}]. & & \end{array}$$

Under the law of mass-action, we model the dynamics of the concentrations over time by the following system of ordinary differential equations:

$$\begin{aligned} \dot{x}_1 &= k_4 x_3 x_5 - k_1 x_1 \\ \dot{x}_2 &= k_5 x_4 x_5 + k_1 x_1 - k_2 x_2 \\ \dot{x}_3 &= k_2 x_2 - k_3 x_3 - k_4 x_3 x_5 \\ \dot{x}_4 &= k_3 x_3 - k_5 x_4 x_5 \\ \dot{x}_5 &= k_6 x_6 - k_4 x_3 x_5 - k_5 x_4 x_5 \\ \dot{x}_6 &= -k_6 x_6 + k_4 x_3 x_5 + k_5 x_4 x_5, \end{aligned}$$

where we write $\dot{x}_* = \frac{dx_*}{dt}$ and omit reference to time t, that is, we write $x_* = x_*(t)$.

Observe that

$$\dot{x}_1 + \dot{x}_2 + \dot{x}_3 + \dot{x}_4 = 0 \quad \text{and} \quad \dot{x}_5 + \dot{x}_6 = 0.$$

It follows that the sums of concentrations $x_1 + x_2 + x_3 + x_4$ and $x_5 + x_6$ are constant over time. This leads to the extra equations

$$x_1 + x_2 + x_3 + x_4 = H, \qquad x_5 + x_6 = T. \tag{1}$$

for some positive total amounts $H, T > 0$.

1.2 Positive steady states

The positive steady states of the system are the solutions to the equations $\dot{x}_1, \ldots, \dot{x}_6 = 0$, constrained by the conservation laws (1). Due to the conservation laws, the equation $\dot{x}_6 = 0$ is fulfilled provided $\dot{x}_5 = 0$ is fulfilled, and similarly, $\dot{x}_4 = 0$ is fulfilled provided $\dot{x}_1 = 0, \dot{x}_2 = 0$, and $\dot{x}_3 = 0$ are fulfilled. Therefore, the equations $\dot{x}_6 = 0$ and $\dot{x}_4 = 0$ can be disregarded.

Consider first the system of equations given by $\dot{x}_1 = 0, \dot{x}_2 = 0, \dot{x}_3 = 0$, and the first equation in (1). That is, consider the system of equations:

$$\begin{aligned} 0 &= k_4 x_3 x_5 - k_1 x_1 \\ 0 &= k_5 x_4 x_5 + k_1 x_1 - k_2 x_2 \\ 0 &= k_2 x_2 - k_3 x_3 - k_4 x_3 x_5 \\ H &= x_1 + x_2 + x_3 + x_4. \end{aligned} \tag{2}$$

This system is linear in x_1, x_2, x_3, x_4 with coefficients involving the rate constants and x_5. We solve it and obtain the following algebraic expressions for x_1, x_2, x_3, x_4 at steady state, depending on the value of x_5 at steady state:

$$x_1 = \frac{k_2k_4k_5Hx_5^2}{(k_1+k_2k_4)k_5x_5^2 + k_1(k_2+k_3)k_5x_5 + k_1k_2k_3} \tag{3}$$

$$x_2 = \frac{k_1(k_4x_5+k_3)k_5Hx_5}{(k_1+k_2k_4)k_5x_5^2 + k_1(k_2+k_3)k_5x_5 + k_1k_2k_3} \tag{4}$$

$$x_3 = \frac{k_1k_2k_5Hx_5}{(k_1+k_2k_4)k_5x_5^2 + k_1(k_2+k_3)k_5x_5 + k_1k_2k_3} \tag{5}$$

$$x_4 = \frac{k_1k_2k_3H}{(k_1+k_2k_4)k_5x_5^2 + k_1(k_2+k_3)k_5x_5 + k_1k_2k_3}. \tag{6}$$

These expressions are positive provided x_5 is positive. We use the second equation in (1) to determine the value at steady state of x_6. Clearly, we have that $x_6 = T - x_5$, which is positive provided $x_5 < T$.

The steady state polynomial. All concentrations are expressed as functions of x_5, and we have not used the equation $\dot{x}_5 = 0$. We can replace the equation $\dot{x}_5 = 0$ by any linear combination of the steady state equations that involves this one. By doing so, the solutions to the equations do not change. We replace it by the equation $\dot{x}_5 + \dot{x}_1 - \dot{x}_4 = 0$. This cancels out the quadratic terms in the equation $\dot{x}_5 = 0$, and we obtain the equation

$$0 = k_6x_6 - k_1x_1 - k_3x_3. \tag{7}$$

Substituting into (7) the values of x_1 and x_3 in (3), (5), and further letting $x_6 = T - x_5$, we obtain that, at steady state, it holds

$$0 = k_6(T - x_5) - \frac{k_1k_2k_4k_5Hx_5^2 + k_1k_2k_3k_5Hx_5}{(k_1+k_2k_4)k_5x_5^2 + k_1(k_2+k_3)k_5x_5 + k_1k_2k_3}. \tag{8}$$

By clearing denominators, the positive solutions to (8) agree with the positive solutions to the polynomial

$$\begin{aligned} p(x_5) &= k_6(T-x_5)((k_1+k_2k_4)k_5x_5^2 + k_1(k_2+k_3)k_5x_5 + k_1k_2k_3) - k_1k_2k_4k_5Hx_5^2 - k_1k_2k_3k_5x_5 \\ &= (k_1+k_2)k_4k_5k_6x_5^3 + (k_1(Hk_2k_4 + k_2k_6 + k_3k_6) - T(k_1+k_2)k_4k_6)k_5x_5^2 \\ &\quad + (k_1k_2k_3(Hk_5+k_6) - Tk_1(k_2+k_3)k_5k_6)x_5 - Tk_1k_2k_3k_6 \end{aligned} \tag{9}$$

The polynomial $p(x_5)$ has degree 3. Any zero of the polynomial between 0 and T corresponds to a positive steady state. From (8), if $x_5 \geq T$ were a positive zero of the polynomial, then we would have 0 equal to a negative number, which is a contradiction. Therefore, any positive solution to the polynomial equation must fulfil that $x_5 < T$ and hence provide a positive steady state.

The polynomial $p(x_5)$ has at most 3 positive roots. We show below in subsection 2.3 that there exist choices of rate constants and total amounts such that $p(x_5)$ indeed has 3 positive roots. Therefore, there exist choices of rate constants and total amounts such that the system has 3 positive steady states.

1.3 Necessary conditions for bistability

Following Descartes' rule of signs, a necessary condition for $p(x_5)$ to have 3 positive roots is that the coefficients of the polynomial have alternating signs. Since the leading coefficient is positive and the independent term is negative, a necessary condition is that the coefficient of degree 2 is negative and the coefficient of degree 1 is positive, that is:

$$k_1(Hk_2k_4 + k_2k_6 + k_3k_6) < T(k_1 + k_2)k_4k_6, \quad k_1k_2k_3(Hk_5 + k_6) > Tk_1(k_2 + k_3)k_5k_6.$$

To derive the necessary condition for bistability stated in the main text, namely $k_1 < k_3$ (in the main text, $k_{s1} < k_{s2}$), we consider equation (8) again. We rewrite it as

$$k_6T = k_6x_5 + \frac{k_1k_2k_4k_5Hx_5^2 + k_1k_2k_3k_5Hx_5}{(k_1 + k_2k_4)k_5x_5^2 + k_1(k_2 + k_3)k_5x_5 + k_1k_2k_3}. \tag{10}$$

If the right-hand side of the equation, call it $\varphi(x_5)$, is an increasing function of x_5 for positive x_5, then for any value of T there will be a unique value of x_5 such that (10) is fulfilled, and hence a unique positive steady state. Since (10) is derived from (7), the function $\varphi(x_5)$ equals $k_6x_5 + k_1x_1 + k_3x_3$, with x_1, x_3 expressed as in (3) and (5).

Clearly, k_6x_5 is increasing in x_5. The derivative of $k_1x_1 + k_3x_3$ with respect to x_5 is

$$\frac{Hk_1k_2^2k_5((k_1 - k_3)k_4k_5x_5^2 + 2\,k_1k_3k_4x_5 + k_1k_3^2)}{((k_1 + k_2k_4)k_5x_5^2 + k_1(k_2 + k_3)k_5x_5 + k_1k_2k_3)^2}.$$

The derivative is not necessarily positive for $x_5 > 0$. However, if $k_1 > k_3$ then the derivative of $\varphi(x_5)$ is positive, implying that φ is an increasing function, and, as a consequence, bistability cannot arise for any value of T.

To summarise, $k_1 > k_3$ implies that there is no bistability, and therefore, a necessary condition for bistability is that $k_3 > k_1$.

1.4 Necessary and sufficient conditions for bistability

The conditions given above are only necessary for bistability, but their fulfilment does not guarantee bistability. We provide here necessary and sufficient conditions on all the parameters of the system for bistability. The parameters include the reaction rate constants and the total amounts. To obtain them, we apply Sturm's Theorem:

Theorem 1 (Sturm)**.** *Let $p(x)$ be a real polynomial. Define recursively the Sturm sequence by*

$$p_0(x) = p(x),\ p_1(x) = p'(x), \quad \text{and} \quad p_{i+1}(x) = -\text{rem}(p_{i-1}, p_i),$$

for $i \geq 1$, where $\text{rem}(p_{i-1}, p_i)$ denotes the reminder of p_{i-1} divided by p_i. The sequence stops when $p_{i+1} = 0$. Let p_m be the last nonzero polynomial.

For $c \in \mathbb{R}$, let $\sigma(c)$ be the number of sign changes in the sequence $p_0(c), \dots, p_m(c)$. Let $a < b$ and assume that neither a nor b are multiple roots of $p(x)$. Then $\sigma(a) - \sigma(b)$ is the number of distinct roots of $p(x)$ in the interval $(a, b]$.

We are interested in the positive roots of the polynomial $p(x) = p(x_5)$ in (9). In this case, $a = 0$, and we need to take b large enough, which is equivalent to considering instead of the sequence $p_0(b), \dots, p_m(b)$, the leading coefficients of the polynomials $p_0, \dots, p_m$. This sequence is written as $p_0(+\infty), \dots, p_m(+\infty)$. Observe that $a = 0$ is not a root of $p(x)$.

According to the theorem, $\sigma(0) - \sigma(+\infty)$ equals the number of distinct positive roots of $p(x)$. Since $\sigma(0), \sigma(+\infty) \geq 0$, the number of distinct roots will be 3, that is, we will have three positive steady states, if and only if $\sigma(0) = 3$ and $\sigma(+\infty) = 0$.

We computed in Maple the Sturm sequence $p_0(x), \dots, p_3(x)$ ($p_4(x) = 0$). For a generic polynomial of degree 3, $p_0(x) = a_0x^3 + a_1x^2 + a_2x + a_3$, the sequence is:

$$\begin{aligned}
p_0(x) &= a_0x^3 + a_1x^2 + a_2x + a_3 \\
p_1(x) &= 3a_0x^2 + 2a_1x + a_2 \\
p_2(x) &= -\frac{6a_0a_2x - 2a_1^2x - 9a_0a_3 - a_1a_2}{9a_0} \\
p_3(x) &= -\frac{9a_0(27a_0^2a_3^2 + 18\,a_0a_1a_2a_3 + 4a_0a_2^3 - 4a_1^3a_3 - a_1^2a_2^2)}{4(3a_0a_2 - a_1^2)^2}.
\end{aligned}$$

In our case, the coefficients are:

$$\begin{aligned}
a_0 &= (k_1 + k_2)k_4k_5k_6 > 0 \\
a_1 &= (k_1(Hk_2k_4 + k_2k_6 + k_3k_6) - T(k_1 + k_2)k_4k_6)k_5 \\
a_2 &= (k_1k_2k_3(Hk_5 + k_6) - Tk_1(k_2 + k_3)k_5k_6) \\
a_3 &= -Tk_1k_2k_3k_6 < 0.
\end{aligned} \tag{11}$$

Hence, $p_0(0) = a_3 < 0$. Therefore, for $\sigma(0) = 3$, we need $p_1(0) > 0, p_2(0) < 0$ and $p_3(0) > 0$. On the other hand,

$$p_0(+\infty) = a_0 > 0 \quad \text{and} \quad p_1(+\infty) = 3a_0 > 0.$$

Therefore, for $\sigma(+\infty) = 0$ we require $p_2(+\infty), p_3(+\infty) > 0$.

The polynomial $p_3(x)$ has degree zero, and hence $p_3(0) = p_3(+\infty)$. Therefore, we are left with 4 conditions on the parameters that fully characterise the region of the parameter space with three steady states, namely $p_1(0), p_3(0), p_2(+\infty) > 0$ and $p_2(0) < 0$. Using that $a_0 > 0$ and $a_3 < 0$, these conditions simplify to the following conditions, where $a_0, \dots, a_3$ need to be substituted by their expressions in (11):

$$\begin{aligned}
a_2 &> 0 & &(p_1(0) > 0) \\
9a_0a_3 + a_1a_2 &< 0 & &(p_2(0) < 0) \\
27a_0^2a_3^2 + 18a_0a_1a_2a_3 - 4a_0a_2^3 + 4a_1^3a_3 - a_1^2a_2^2 &< 0 & &(p_3(0) > 0) \\
-6a_0a_2 + 2a_1^2 &> 0 & &(p_2(+\infty) > 0).
\end{aligned}$$

That is, the system has three positive steady states if and only if the 4 inequalities above are satisfied.

2 The core model for n hybrid HKs competing for the same Hpt

2.1 Model description

We study here the core system consisting of n hybrid HKs competing for the same Hpt. We call such a system an *nHK-Hpt system* for short. In this case, there are n hybrid HKs, which we denote by HK^i, for $i = 1, \dots, n$, and we use subindices 00, P0, 0P, PP to denote the phosphorylation state of each of them.

The set of reactions given in the previous subsection are reproduced for the n hybrid HKs. That is, for $i = 1, \dots, n$, the reactions for the transfer of phosphate group are as follows:

$$\mathrm{HK}^i_{00} \xrightarrow{k_{i,1}} \mathrm{HK}^i_{\mathrm{P}0} \xrightarrow{k_{i,2}} \mathrm{HK}^i_{0\mathrm{P}} \xrightarrow{k_{i,3}} \mathrm{HK}^i_{\mathrm{PP}} \qquad \mathrm{HK}^i_{0\mathrm{P}} + \mathrm{Hpt} \xrightarrow{k_{i,4}} \mathrm{HK}^i_{00} + \mathrm{Hpt}_{\mathrm{P}}$$

$$\mathrm{HK}^i_{\mathrm{PP}} + \mathrm{Hpt} \xrightarrow{k_{i,5}} \mathrm{HK}^i_{\mathrm{P}0} + \mathrm{Hpt}_{\mathrm{P}}$$

and there is further the dephosphorylation reaction

$$\mathrm{Hpt}_{\mathrm{P}} \xrightarrow{k_6} \mathrm{Hpt}.$$

We denote the concentration of the species as follows:

$$x_{i,1} := [\mathrm{HK}^i_{00}] \qquad x_{i,2} := [\mathrm{HK}^i_{\mathrm{P}0}] \qquad x_{i,3} := [\mathrm{HK}^i_{0\mathrm{P}}] \qquad x_{i,4} := [\mathrm{HK}^i_{\mathrm{PP}}]$$
$$x_5 := [\mathrm{Hpt}] \qquad x_6 := [\mathrm{Hpt}_{\mathrm{P}}],$$

for $i = 1, \dots, n$. Under the law of mass-action, we model the dynamics of the concentrations over time by the following system of ordinary differential equations:

$$\begin{aligned}
\dot{x}_{i,1} &= k_{i,4}x_{i,3}x_5 - k_{i,1}x_{i,1} \\
\dot{x}_{i,2} &= k_{i,5}x_{i,4}x_5 + k_{i,1}x_{i,1} - k_{i,2}x_{i,2} \\
\dot{x}_{i,3} &= k_{i,2}x_{i,2} - k_{i,3}x_{i,3} - k_{i,4}x_{i,3}x_5 \\
\dot{x}_{i,4} &= k_{i,3}x_{i,3} - k_{i,5}x_{i,4}x_5 \\
\dot{x}_5 &= k_6x_6 - \sum_{j=1}^{n}(k_{j,4}x_{j,3}x_5 + k_{j,5}x_{j,4}x_5) \\
\dot{x}_6 &= -k_6x_6 + \sum_{j=1}^{n}(k_{j,4}x_{j,3}x_5 + k_{j,5}x_{j,4}x_5),
\end{aligned}$$

for $i = 1, \dots, n$. The system has $n + 1$ conservation laws. Namely, for $i = 1, \dots, n$ we have

$$x_{i,1} + x_{i,2} + x_{i,3} + x_{i,4} = H_i \tag{12}$$

for some $H_i > 0$, and for $T > 0$,

$$x_5 + x_6 = T. \tag{13}$$

2.2 Positive steady states

The positive steady states of the system are the solutions to the equations $\dot{x}_{i,1} = 0, \dot{x}_{i,2} = 0, \dot{x}_{i,3} = 0, \dot{x}_{i,4} = 0$, for $i = 1, \dots, n$, together with $\dot{x}_5 = 0, \dot{x}_6 = 0$, constrained by the conservation laws (12) and (13). We reason as in the previous section to disregard the steady state equations $\dot{x}_6 = 0$ and $\dot{x}_{i,4} = 0$, for $i = 1, \dots, n$.

Using the equations $\dot{x}_{i,1} = 0, \dot{x}_{i,2} = 0, \dot{x}_{i,3} = 0$ and (12), namely

$$\begin{aligned}
0 &= k_{i,4}x_{i,3}x_5 - k_{i,1}x_{i,1} \\
0 &= k_{i,5}x_{i,4}x_5 + k_{i,1}x_{i,1} - k_{i,2}x_{i,2} \\
0 &= k_{i,2}x_{i,2} - k_{i,3}x_{i,3} - k_{i,4}x_{i,3}x_5 \\
H_i &= x_{i,1} + x_{i,2} + x_{i,3} + x_{i,4},
\end{aligned} \tag{14}$$

we obtain the algebraic expressions for $x_{i,1}, x_{i,2}, x_{i,3}, x_{i,4}$ at steady state, depending on the value of x_5 at steady state, analogous to the expressions (3)-(6):

$$x_{i,1} = \frac{k_{i,2}k_{i,4}k_{i,5}H_i x_5^2}{(k_{i,1}+k_{i,2}k_{i,4})k_{i,5}x_5^2 + k_{i,1}(k_{i,2}+k_{i,3})k_{i,5}x_5 + k_{i,1}k_{i,2}k_{i,3}} \tag{15}$$

$$x_{i,2} = \frac{k_{i,1}(k_{i,4}x_5 + k_{i,3})k_{i,5}H_i x_5}{(k_{i,1}+k_{i,2}k_{i,4})k_{i,5}x_5^2 + k_{i,1}(k_{i,2}+k_{i,3})k_{i,5}x_5 + k_{i,1}k_{i,2}k_{i,3}} \tag{16}$$

$$x_{i,3} = \frac{k_{i,1}k_{i,2}k_{i,5}H_i x_5}{(k_{i,1}+k_{i,2}k_{i,4})k_{i,5}x_5^2 + k_{i,1}(k_{i,2}+k_{i,3})k_{i,5}x_5 + k_{i,1}k_{i,2}k_{i,3}} \tag{17}$$

$$x_{i,4} = \frac{k_{i,1}k_{i,2}k_{i,3}H_i}{(k_{i,1}+k_{i,2}k_{i,4})k_{i,5}x_5^2 + k_{i,1}(k_{i,2}+k_{i,3})k_{i,5}x_5 + k_{i,1}k_{i,2}k_{i,3}}. \tag{18}$$

These expressions are positive provided x_5 is positive. From (13) we have that $x_6 = T - x_5$, which is positive provided $x_5 < T$.

We replace the steady state equation $\dot{x}_5 = 0$ by $\dot{x}_5 + \sum_{i=1}^{n}(\dot{x}_{i,1} - \dot{x}_{i,4}) = 0$, and we obtain the equation

$$0 = k_6 x_6 - \sum_{i=1}^{n}(k_{i,1}x_{i,1} + k_{i,3}x_{i,3}). \tag{19}$$

Substituting into (19) the values of $x_{i,1}$ and $x_{i,3}$ in (15), (17), and further letting $x_6 = T - x_5$, we obtain that, at steady state, it holds

$$0 = k_6(T - x_5) - \sum_{i=1}^{n} \frac{k_{i,1}k_{i,2}k_{i,4}k_{i,5}H_i x_5^2 + k_{i,1}k_{i,2}k_{i,3}k_{i,5}H_i x_5}{(k_{i,1}+k_{i,2}k_{i,4})k_{i,5}x_5^2 + k_{i,1}(k_{i,2}+k_{i,3})k_{i,5}x_5 + k_{i,1}k_{i,2}k_{i,3}}. \tag{20}$$

By clearing denominators, that is, by multiplying equation (20) by

$$\prod_{i=1}^{n}(k_{i,1}+k_{i,2}k_{i,4})k_{i,5}x_5^2 + k_{i,1}(k_{i,2}+k_{i,3})k_{i,5}x_5 + k_{i,1}k_{i,2}k_{i,3},$$

we obtain a polynomial of degree $2n+1$ in x_5. Any zero of the polynomial, that lies between 0 and T, corresponds to a positive steady state. We argue again that, if $x_5 \geq T$ were a positive zero of the polynomial, equation (20) would give a contradiction. Therefore, any positive solution to the polynomial equation must fulfill that $x_5 < T$ and hence provide a positive steady state.

2.3 Existence $2n + 1$ positive steady states.

We have shown that the positive steady states of the nHK-Hpt system are determined by the positive solutions to (20). Solving for the positive solutions to this equation is equivalent to solving for the positive solutions to a polynomial of degree $2n + 1$. By the fundamental theorem of algebra, a polynomial of degree $2n + 1$ has $2n + 1$ complex roots counted with multiplicity. Therefore, such a polynomial can *at most* have $2n+1$ distinct positive real roots.

We show in this section that there exist choices of rate constants k_* and total amounts H_i, T such that the polynomial has exactly $2n + 1$ distinct positive real roots. As a consequence, this proves that the nHK-Hpt system admits $2n + 1$ positive steady states for some choice of rate constants and total amounts.

The proof consists of a series of simplifications and constructions. A key ingredient of the proof is the following theorem:

Theorem 2 (Kurtz [5]). *Let $m \geq 1$ and let $p(x) = x^{2m+1} - c_1 x^{2m} + c_2 x^{2m-1} + \cdots + c_{2m} x - c_{2m+1}$ be a polynomial of odd degree $2m+1$ and with $c_i \geq 0$ for all i. Let $c_0 = 1$. If*

$$c_i^2 - 4c_{i-1}c_{i+1} > 0 \tag{21}$$

for all $i = 1, \ldots, 2m$, then $p(x)$ has $2m+1$ distinct positive real roots.

For clarity, we provide the main arguments of our proof here in the form of lemmas, which are proven in the next section.

First of all observe that the steady states of the system are invariant by multiplication of all rate constants by some scalar $\lambda > 0$. Therefore, we assume that $k_6 = 1$. For simplicity we write x for x_5.

We let

$$\alpha_{i,1} = k_{i,1}k_{i,2}k_{i,4}k_{i,5}H_i \tag{22}$$
$$\alpha_{i,2} = k_{i,1}k_{i,2}k_{i,3}k_{i,5}H_i \tag{23}$$
$$\alpha_{i,3} = (k_{i,1} + k_{i,2})k_{i,4}k_{i,5} \tag{24}$$
$$\alpha_{i,4} = k_{i,1}(k_{i,2} + k_{i,3})k_{i,5} \tag{25}$$
$$\alpha_{i,5} = k_{i,1}k_{i,2}k_{i,3}, \tag{26}$$

such that we write

$$\frac{k_{i,1}k_{i,2}k_{i,4}k_{i,5}H_i x^2 + k_{i,1}k_{i,2}k_{i,3}k_{i,5}H_i x}{(k_{i,1} + k_{i,2}k_{i,4})k_{i,5}x_5^2 + k_{i,1}(k_{i,2} + k_{i,3})k_{i,5}x_5 + k_{i,1}k_{i,2}k_{i,3}} = \frac{\alpha_{i,1}x^2 + \alpha_{i,2}x}{\alpha_{i,3}x^2 + \alpha_{i,4}x + \alpha_{i,5}}.$$

Lemma 1. *For any positive values $\alpha_{i,1}, \ldots, \alpha_{i,5} > 0$, there exist $k_{i,1}, \ldots, k_{i,5} > 0$ and $H_i > 0$ such that (22)-(26) are fulfilled.*

As a consequence of Lemma 1, there exist rate constants and total amounts such that (20) holds if we can find $\alpha_{i,1}, \ldots, \alpha_{i,5} > 0$ such that

$$0 = -T + x + \sum_{i=1}^{n} \frac{\alpha_{i,1}x^2 + \alpha_{i,2}x}{\alpha_{i,3}x^2 + \alpha_{i,4}x + \alpha_{i,5}}. \tag{27}$$

With this notation, we want to determine the positive real roots of the polynomial obtained by clearing denominators in (27):

$$q(x) = (x - T)\prod_{i=1}^{n}(\alpha_{i,3}x^2 + \alpha_{i,4}x + \alpha_{i,5}) + \sum_{i=1}^{n}\Big((\alpha_{i,1}x^2 + \alpha_{i,2}x)\prod_{j\neq i}(\alpha_{j,3}x^2 + \alpha_{j,4}x + \alpha_{j,5})\Big). \tag{28}$$

The coefficient of degree $2n+1$ of $q(x)$ is $\prod_{i=1}^{n}\alpha_{i,3}$ and the independent term of $q(x)$ is $-T\prod_{i=1}^{n}\alpha_{i,5}$. We would like to apply Theorem 2 to such a polynomial. To this end, the coefficients of monomials with even degree should be negative and those of odd degree should be positive. The latter is guaranteed if $\alpha_{i,4} = 0$, while the former if $\alpha_{i,1} = 0$. Setting these two constants to zero, for $i = 1, \ldots, n$, does not change the degree of the polynomial.

By the continuity of the isolated roots of a polynomial as functions of the coefficients of the polynomial, if we can find $\alpha_{i,2}, \alpha_{i,3}, \alpha_{i,5} > 0$ such that with $\alpha_{i,4} = 0, \alpha_{i,1} = 0$, the polynomial $q(x)$ has $2n+1$ distinct positive real roots, then for $\alpha_{i,4}, \alpha_{i,1}$ small enough, the polynomial $q(x)$ still has $2n+1$ distinct positive real roots.

This is what we do next. We set $\alpha_{i,4} = 0, \alpha_{i,1} = 0$ and further $\alpha_{i,3} = 1$ for all $i = 1, \dots, n$, and $T = 1$. To ease the notation, we write $a_i = \alpha_{i,2}$ and $b_i = \alpha_{i,5}$, such that the polynomial of interest is

$$p(x) = (x-1)\prod_{i=1}^{n}(x^2+b_i) + \sum_{i=1}^{n}\Big(a_i x \prod_{j\neq i}(x^2+b_j)\Big). \tag{29}$$

We denote by $[n] = \{1, \dots, n\}$. In the next lemma we describe the coefficients of $p(x)$. The form of the coefficients depends on the parity of the degree of the coefficient. Therefore the coefficients take two different forms, one for even subindices, that is $i = 2k$, and one for odd subindices, that is $i = 2k+1$.

Lemma 2. *Let the polynomial* (29) *be written as* $p(x) = c_0 x^{2n+1} + c_1 x^{2n} + c_2 x^{2n-1} + \dots + c_{2n} x + c_{2n+1}$. *Then it holds:*

$$c_{2k+1} = -\sum_{\{j_1,\dots,j_k\}\subseteq[n]}\prod_{\ell=1}^{k} b_{j_\ell}$$

$$c_{2k} = \sum_{\{j_1,\dots,j_k\}\subseteq[n]}\prod_{\ell=1}^{k} b_{j_\ell} + \sum_{i=1}^{n} a_i \sum_{\{j_1,\dots,j_{k-1}\}\subseteq[n]\setminus\{i\}}\prod_{\ell=1}^{k-1} b_{j_\ell}$$

for $k = 0, \dots, n$, *with the convention that the sum and the product over the empty set equals* 1.

For example, for $n = 1$ we have

$$p(x) = x^3 - x^2 + (a_1 + b_1)x - b_1,$$

while for $n = 2$ we have

$$p(x) = x^5 - x^4 + (a_1 + a_2 + b_1 + b_2)x^3 - (b_1 + b_2)x^2 + (a_1 b_2 + a_2 b_1 + b_1 b_2)x - b_1 b_2.$$

All that is left is to show that we can find b_i, a_i such that the polynomial $p(x)$ satisfies the inequalities in Theorem 2. This is the content of the following lemmas. We provide in Lemma 3 a choice of constants b_i such that the inequalities (21) are fulfilled for even indices i, that is, $i = 2k$ for some k. In Lemma 4 we provide a choice of constants a_i such that the inequalities (21) are fulfilled for odd indices i, that is, $i = 2k+1$ for some k.

Lemma 3. *Fix arbitrary* $a_1, \dots, a_n > 0$ *and define* $b_i = \frac{a_i^2}{4}$ *for* $i = 1, \dots, n$. *Then,*

$$c_{2k}^2 - 4c_{2k-1}c_{2k+1} > 0$$

for all $k = 1, \dots, n$.

Lemma 4. *Let* $M > 0$ *and* $a_n > 0$. *For* $i = 1, \dots, n$, *define* $a_i = \frac{a_n}{M^{i-1}}$ *and* $b_i = \frac{a_i^2}{4}$. *Then, for* M *large enough and* a_n *small enough, it holds*

$$c_{2k+1}^2 - 4c_{2k}c_{2k+2} > 0$$

for all $k = 0, \dots, n-1$.

We are ready to prove the main result on the number of positive steady states.

Theorem 3. *For any $n \geq 1$, there exists a choice of rate constants $k_6 > 0$, $k_{i,1}, k_{i,2}, k_{i,3}, k_{i,4} > 0$ and total amounts $T, H_i > 0$, for $i = 1, \dots, n$, such that the nHK-Hpt system has $2n+1$ distinct positive steady states.*

Proof. Pick $a_n, M > 0$ and define b_i, a_i, for $i = 1, \dots, n$, as in Lemma 4. By lemmas 4 and 3, by choosing M large enough and a_n small enough, the inequalities (21) hold. We set $\alpha_{i,2} = a_i, \alpha_{i,5} = b_i, \alpha_{i,3} = 1$, for $i = 1, \dots, n$, and $T = 1$. Then $p(x)$ in (29) has $2n+1$ distinct positive real roots. We choose $\alpha_{i,1}, \alpha_{i,4} > 0$ small enough such that the polynomial $q(x)$ in (28) has $2n+1$ distinct positive real roots. We set $k_6 = 1$. By construction, any choice $k_{i,1}, k_{i,2}, k_{i,3}, k_{i,4} > 0$ and $H_i > 0$ such that (22)-(26) are fulfilled provides a set of parameters with $2n+1$ distinct positive steady states. Such a choice exists by Lemma 1. □

Observe that the proof is constructive. It gives a procedure to find sets of parameters with the maximal number of steady states. The several checks that the proof requires are easily implemented using most available mathematical software to solve equations (e.g. Maple, Mathematica).

In general, we have observed that given any polynomial $u(x)$ with $2n+1$ distinct positive real roots we can find a_i, b_i such that the coefficients of $u(x)$ agree with c_i in Lemma 2, even if $u(x)$ does not fulfill the conditions of Theorem 2. Such a_i, b_i can be found using mathematical software.

2.4 n unstable steady states

In the subsection we show that, considering the $2n+1$ steady states ordered increasingly by their value $x = x_5$, then the steady states number $2, 4, \dots, 2n$ are unstable relative to the stoichiometric compatibility class they belong to, that is, relative to the invariant subspaces described by the conservation laws (12) and (13).

Since the nHK-Hpt system has $4n+2$ variables and $n+1$ conservation laws, the Jacobian of f in $\dot{x} = f(x)$ always has $n+1$ zero eigenvalues. The remaining $3n+1$ eigenvalues (which could include zero) have corresponding eigenvectors in the stoichiometric subspace and dictate the dynamics around the steady state and within the stoichiometric compatibility class. If the steady state is locally stable relative to the stoichiometric compatibility class, then the product of these $3n+1$ eigenvalues has sign $(-1)^{3n+1}$. Therefore, if the sign of the product of these eigenvalues is $(-1)^{3n}$, then the steady state is necessarily locally unstable relative to the stoichiometric compatibility class. We argue in the proof of the next theorem that this is the case for the steady states in even position $2, 4, \dots, 2n$.

Theorem 4. *The $2, 4, \dots, 2n$-th steady states are unstable relative to the stoichiometric compatibility class.*

Proof. We order the variables of the system as $x_{1,1}, x_{1,2}, x_{1,3}, x_{1,4}, \dots, x_{n,1}, x_{n,2}, x_{n,3}, x_{n,4}, x_6, x_5$. It follows from [6, Prop. 5.3] that the product of the $3n+1$ eigenvalues of the Jacobian with eigenvectors in the stoichiometric space agrees with the determinant of the Jacobian of the function $g\colon \mathbb{R}^{4n+2} \to \mathbb{R}^{4n+2}$ where

$$
\begin{aligned}
g_{4(i-1)+1}(x) &= x_{i,1} + x_{i,2} + x_{i,3} + x_{i,4} - H_i \\
g_{4(i-1)+2}(x) &= k_{i,5} x_{i,4} x_5 + k_{i,1} x_{i,1} - k_{i,2} x_{i,2} \\
g_{4(i-1)+3}(x) &= k_{i,2} x_{i,2} - k_{i,3} x_{i,3} - k_{i,4} x_{i,3} x_5 \\
g_{4(i-1)+4}(x) &= x_5 + k_{i,3} x_{i,3} - k_{i,5} x_{i,4},
\end{aligned}
$$

for $i = 1, \dots, n$ and

$$g_{4n+1}(x) = x_5 + x_6 - T,$$
$$g_{4n+2}(x) = k_6 x_6 - \sum_{j=1}^{n} (k_{j,4} x_{j,3} + k_{j,5} x_{j,4}) x_5.$$

We now apply the method described in [2] (see the Electronic Supplementary Material), to determine the sign of the determinant of the Jacobian of g from iterative eliminations. One can check that the expressions in (15)-(18) are obtained from iteratively eliminating $x_{i,1}, \dots, x_{i,4}$ from the equations $g_{4(i-1)+1}(x) = \cdots = g_{4(i-1)+4}(x) = 0$, which are equivalent to (14), and which correspond to the conservation law together with $\dot{x}_{i,2} = \dot{x}_{i,3} = \dot{x}_{i,4} = 0$.

Let $q(x_5)$ be the polynomial obtained after clearing denominators in (20). Then, by [2], the sign of the determinant of the Jacobian of g at a steady state agrees with the sign of the derivative of $q(x_5)$, $q'(x_5)$, times $(-1)^{3n}$. Therefore, if $q'(x_5)$ is positive, then the corresponding steady state is locally unstable. Since $q(0)$ is positive, the first real root of $q(x_5)$ has negative derivative, and then the signs alternate. Therefore, the steady states corresponding to the $2, 4, \dots, 2n$-th roots are locally unstable relatively to the stoichiometric compatibility class. □

3 Multiple phosphorelays with a common RR

3.1 The core model for M hybrid phosphorelays competing for an RR

In the previous section we showed that unlimited multistationarity arises by increasing the number of hybrid HKs that compete for the same Hpt. In this section we show that the same statement holds when the competition occurs at the level of the response regulator RR. For example, we show that the system consisting of two core hybrid phosphorelays, complete with their own Hpt, competing for the same RR can have up to 9 steady states. This system consists of two 1HK-Hpt systems, where the two independent Hpt's donate their phosphate group to the same RR.

We study such a system with full generality. We allow each of the phosphorelays to have multiple hybrid HKs, as studied in the previous sections. For example, consider a system with two His-containing phosphotransfer proteins Hpt^1 and Hpt^2 that transfer the phosphate group to the same RR. Assume, for instance, that Hpt^1 receives the phosphate group from two hybrid HKs, $\mathrm{HK}^{1,1}$ and $\mathrm{HK}^{1,2}$, and that Hpt^2 receives the phosphate group from one hybrid HK, $\mathrm{HK}^{2,1}$. The first upper index of $\mathrm{HK}^{i,j}$ indicates the Hpt index, and the second index indicates the index of the HK in the nHK-Hpt subsystem. By using the notation introduced in the previous section to denote phosphorylated sites, the reactions of this example system are as follows:

(i) Reactions within each HK:

$$\mathrm{HK}^{1,1}_{00} \xrightarrow{k_{1,1,1}} \mathrm{HK}^{1,1}_{\mathrm{P}0} \xrightarrow{k_{1,1,2}} \mathrm{HK}^{1,1}_{0\mathrm{P}} \xrightarrow{k_{1,1,3}} \mathrm{HK}^{1,1}_{\mathrm{PP}}$$

$$\mathrm{HK}^{1,2}_{00} \xrightarrow{k_{1,2,1}} \mathrm{HK}^{1,2}_{\mathrm{P}0} \xrightarrow{k_{1,2,2}} \mathrm{HK}^{1,2}_{0\mathrm{P}} \xrightarrow{k_{1,2,3}} \mathrm{HK}^{1,2}_{\mathrm{PP}}$$

$$\mathrm{HK}^{2,1}_{00} \xrightarrow{k_{2,1,1}} \mathrm{HK}^{2,1}_{\mathrm{P}0} \xrightarrow{k_{2,1,2}} \mathrm{HK}^{2,1}_{0\mathrm{P}} \xrightarrow{k_{2,1,3}} \mathrm{HK}^{2,1}_{\mathrm{PP}}$$

(ii) Phosphotransfer from $\text{HK}^{1,1}$ and $\text{HK}^{1,2}$ to Hpt^1, and phosphotransfer from $\text{HK}^{2,1}$ to Hpt^2:

$$\text{HK}^{1,1}_{0\text{P}} + \text{Hpt}^1 \xrightarrow{k_{1,1,4}} \text{HK}^{1,1}_{00} + \text{Hpt}^1_{\text{P}} \qquad \text{HK}^{1,1}_{\text{PP}} + \text{Hpt}^1 \xrightarrow{k_{1,1,5}} \text{HK}^{1,1}_{\text{P}0} + \text{Hpt}^1_{\text{P}}$$

$$\text{HK}^{1,2}_{0\text{P}} + \text{Hpt}^1 \xrightarrow{k_{1,2,4}} \text{HK}^{1,2}_{00} + \text{Hpt}^1_{\text{P}} \qquad \text{HK}^{1,2}_{\text{PP}} + \text{Hpt}^1 \xrightarrow{k_{1,2,5}} \text{HK}^{1,2}_{\text{P}0} + \text{Hpt}^1_{\text{P}}$$

$$\text{HK}^{2,1}_{0\text{P}} + \text{Hpt}^2 \xrightarrow{k_{2,1,4}} \text{HK}^{2,1}_{00} + \text{Hpt}^2_{\text{P}} \qquad \text{HK}^{2,1}_{\text{PP}} + \text{Hpt}^2 \xrightarrow{k_{2,1,5}} \text{HK}^{2,1}_{\text{P}0} + \text{Hpt}^2_{\text{P}}$$

(iii) Phosphotransfer from Hpt^1 and Hpt^2 to RR:

$$\text{Hpt}^1_{\text{P}} + \text{RR} \xrightarrow{k_{1,6}} \text{Hpt}^1 + \text{RR}_{\text{P}} \qquad \text{Hpt}^2_{\text{P}} + \text{RR} \xrightarrow{k_{2,6}} \text{Hpt}^2 + \text{RR}_{\text{P}}$$

(iv) Dephosphorylation reaction for RR:

$$\text{RR}_{\text{P}} \xrightarrow{k_7} \text{RR}.$$

3.2 Multiple positive steady states

In general, consider M multiple hybrid phosphorelays competing for the same RR. We let n_i be the number of hybrid HKs of the ith system. That is, each phosphorelay consists of a n_iHK-Hpt system with a further phosphotransfer to RR.

In the above example, we have $M = 2$ and $n_1 = 2, n_2 = 1$. We let $\text{HK}^{i,j}$ denote the jth hybrid kinase of the ith multiple HK-Hpt system, where j runs from 1 to n_i, and we let Hpt^i denote the Hpt of the system. We denote the reaction rates by $k_{i,j,*}$ for those involving $\text{HK}^{i,j}$, the dephosphorylation reaction for Hpt^i by $k_{i,6}$ and the dephosphorylation reaction for RR by k_7. The system has conserved total amounts of $\text{HK}^{i,j}$, Hpt^i and RR.

We denote the concentration of the species as follows:

$$\begin{array}{rrrr} x_{i,j,1} := [\text{HK}^{i,j}_{00}] & x_{i,j,2} := [\text{HK}^{i,j}_{\text{P}0}] & x_{i,j,3} := [\text{HK}^{i,j}_{0\text{P}}] & x_{i,j,4} := [\text{HK}^{i,j}_{\text{PP}}] \\ x_{i,5} := [\text{Hpt}^i] & x_{i,6} := [\text{Hpt}^i_{\text{P}}], & x_7 = [\text{RR}], & x_8 = [\text{RR}_{\text{P}}] \end{array}$$

for $i = 1, \dots, M$, $j = 1, \dots, n_i$.

Under the law of mass-action, we model the dynamics of the concentrations over time by

the following system of ordinary differential equations:

$$\begin{aligned}
\dot{x}_{i,j,1} &= k_{i,j,4}x_{i,j,3}x_{i,5} - k_{i,j,1}x_{i,j,1}\\
\dot{x}_{i,j,2} &= k_{i,j,5}x_{i,j,4}x_{i,5} + k_{i,j,1}x_{i,j,1} - k_{i,j,2}x_{i,j,2}\\
\dot{x}_{i,j,3} &= k_{i,j,2}x_{i,j,2} - k_{i,j,3}x_{i,j,3} - k_{i,j,4}x_{i,j,3}x_{i,5}\\
\dot{x}_{i,j,4} &= k_{i,j,3}x_{i,j,3} - k_{i,5}x_{i,j,4}x_{i,5}\\
\dot{x}_{i,5} &= k_{i,6}x_{i,6}x_7 - \sum_{\ell=1}^{n_i}(k_{i,\ell,4}x_{i,\ell,3} + k_{i,\ell,5}x_{i,\ell,4})x_{i,5}\\
\dot{x}_{i,6} &= -k_{i,6}x_{i,6}x_7 + \sum_{\ell=1}^{n_i}(k_{i,\ell,4}x_{i,\ell,3} + k_{i,\ell,5}x_{i,\ell,4})x_{i,5},\\
\dot{x}_7 &= k_7x_8 - \sum_{\ell=1}^{M}k_{\ell,6}x_{\ell,6}x_7\\
\dot{x}_8 &= -k_7x_8 + \sum_{\ell=1}^{M}k_{\ell,6}x_{\ell,6}x_7,
\end{aligned}$$

for $i = 1,\dots,M$, $j = 1,\dots,n_i$. Further, we have the following conservation law equations:

$$x_{i,j,1} + x_{i,j,2} + x_{i,j,3} + x_{i,j,4} = H_{i,j}, \qquad x_{i,5} + x_{i,6} = T_i, \qquad x_7 + x_8 = R.$$

For each i,j, solving for $\dot{x}_{i,j,1} = 0, \dot{x}_{i,j,2} = 0, \dot{x}_{i,j,3} = 0$ and the conservation law with $H_{i,j}$, we get equalities analogous to (15)-(18), where we replace the subindex i by the pair i,j. The steady state concentrations $x_{i,j}$ are expressed in terms of $x_{i,5}$ and are positive provided $x_{i,5}$ is positive.

We further have $x_{i,6} = T_i - x_{i,5}$. The equation $\dot{x}_{i,5} + \sum_{j=1}^{n_i}(\dot{x}_{i,j,1} - \dot{x}_{i,j,4}) = 0$ replaces $\dot{x}_{i,5} = 0$ and leads to the steady state equation

$$0 = k_{i,6}x_7x_{i,6} - \sum_{j=1}^{n_i}(k_{i,j,1}x_{i,j,1} + k_{i,j,3}x_{i,j,3}), \tag{30}$$

which transforms, as in (20), into the equation

$$0 = k_{i,6}x_7(T_i - x_{i,5}) - \sum_{j=1}^{n_i}\frac{k_{i,j,1}k_{i,j,2}k_{i,j,5}H_{i,j}x_{i,5}(k_{i,j,4}x_{i,5} + k_{i,j,3})}{(k_{i,j,1}+k_{i,j,2})k_{i,j,4}k_{i,j,5}x_{i,5}^2 + k_{i,j,1}(k_{i,j,2}+k_{i,j,3})k_{i,j,5}x_{i,5} + k_{i,j,1}k_{i,j,2}k_{i,j,3}}, \tag{31}$$

for $i = 1,\dots,M$.

Finally, using the total amount R and $\dot{x}_7 = 0$, we obtain:

$$\begin{aligned}
x_7 &= \frac{k_7R}{k_7 + \sum_{\ell=1}^{M}k_{\ell,6}x_{\ell,6}} = \frac{k_7R}{k_7 + \sum_{\ell=1}^{M}k_{\ell,6}(T_\ell - x_{\ell,5})}\\
x_8 &= \frac{R\sum_{\ell=1}^{M}k_{\ell,6}x_{\ell,6}}{k_7 + \sum_{\ell=1}^{M}k_{\ell,6}x_{\ell,6}} = \frac{R\sum_{\ell=1}^{M}k_{\ell,6}(T_\ell - x_{\ell,5})}{k_7 + \sum_{\ell=1}^{M}k_{\ell,6}(T_\ell - x_{\ell,5})}.
\end{aligned}$$

Observe that both x_7, x_8 are positive provided $x_{i,5} < T_i$ for all i. By substituting the expression for x_7 into (31), we deduce that the steady states of the system are found by finding the positive solutions to (31) in $x_{1,5},\dots,x_{M,5}$. The value at steady state of the other concentrations are found using the expressions above. Recall that, as seen in the previous section, a positive solution to (31) must satisfy $x_{i,5} < T_i$ and hence $x_{i,6}$ is positive.

Theorem 5. *Consider the system with M multiple hybrid phosphorelays competing for the same RR, and let n_i be the number of hybrid HKs of the ith system. Then there exists a choice of rate constants and total amounts such that the system has $\prod_{i=1}^{M} 2n_i + 1$ positive steady states.*

Proof. For each $i = 1, \dots, M$, fix parameters $k_{i,j,1}, \dots, k_{i,j,5}, H_{i,j}, T_i$ such that the n_iHK-Hpt system has $2n_i + 1$ steady states, when the dephosphorylation rate constant for Hpt^i in the isolated system is set to one. By Theorem 3 such a choice exists. With this choice, let

$$A_i := \sum_{j=1}^{n_i} \frac{k_{i,j,1}k_{i,j,2}k_{i,j,5}H_{i,j}x_{i,5}(k_{i,j,4}x_{i,5} + k_{i,j,3})}{(k_{i,j,1} + k_{i,j,2})k_{i,j,4}k_{i,j,5}x_{i,5}^2 + k_{i,j,1}(k_{i,j,2} + k_{i,j,3})k_{i,j,5}x_{i,5} + k_{i,j,1}k_{i,j,2}k_{i,j,3}},$$

which only depends on $x_{i,5}$. Consider the map

$$\begin{aligned} \varphi \colon \mathbb{R} \times \mathbb{R}^M &\to \mathbb{R}^M \\ (S, x_{1,5}, \dots, x_{M,5}) &\mapsto \left(\frac{(T_i - x_{i,5})}{1 + S\sum_{\ell=1}^{M}(T_\ell - x_{\ell,5})} - A_i \right)_{i=1,\dots,M}. \end{aligned}$$

For $S = 0$, the ith component of $\varphi(0, x_{1,5}, \dots, x_{M,5}) = 0$ is $T_i - x_{i,5} - A_i = 0$, which is the steady state equation (20) for the i-th system $\mathrm{HK}^{i,*}$ and Hpt^i with our choice of rate constants. By the above choice, such equation has $2n_i + 1$ positive solutions. Since the ith component of $\varphi(0, x_{1,5}, \dots, x_{M,5})$ depends only on $x_{i,5}$, it follows that the equation $\varphi(0, x_{1,5}, \dots, x_{M,5}) = 0$ has $\prod_{i=1}^{M} 2n_i + 1$ positive solutions.

The Jacobian of $\varphi(0, x_{1,5}, \dots, x_{M,5})$ at a solution $(x^*_{1,5}, \dots, x^*_{M,5})$ is a diagonal matrix, whose ith entry is

$$\frac{\partial}{\partial x_{i,5}} \varphi_i(0, x_{1,5}, \dots, x_{M,5}) = \frac{\partial}{\partial x_{i,5}}(T_i - x_{i,5} - A_i)$$

evaluated at $x^*_{i,5}$. Since for each i, our set of positive solutions to $T_i - x_{i,5} - A_i = 0$ is maximal in number, there are no multiple solutions and such a derivative is nonzero.

Therefore, the Jacobian of $\varphi(0, x_{1,5}, \dots, x_{M,5})$ at a solution is nonsingular. This allows us to apply the implicit function theorem to ensure that for $S > 0$ small enough, the equation $\varphi(0, x_{1,5}, \dots, x_{M,5}) = 0$ has precisely $\prod_{i=1}^{M} 2n_i + 1$ positive solutions.

Fix any such $S > 0$ and define $k_{i,6} = S$, $R = \frac{1}{S}$, and $k_7 = 1$. Then

$$\frac{(T_i - x_{i,5})}{1 + S\sum_{\ell=1}^{M}(T_\ell - x_{\ell,5})} = \frac{k_7 k_{i,6} R(T_i - x_{i,5})}{k_7 + \sum_{\ell=1}^{M} k_{\ell,6}(T_\ell - x_{\ell,5})},$$

which corresponds to the steady state equation (31). With this choice, the system has $\prod_{i=1}^{M} 2n_i + 1$ positive steady states. □

The proof gives a constructive way to find sets of parameters with $\prod_{i=1}^{M} 2n_i + 1$ positive steady states. We fix parameters for the individual nHK-Hpt systems that have $2n_i + 1$ steady states, when the dephosphorylation rate constant for Hpt^i in the isolated system is set to one. Then, let $k_7 = 1$, $k_{i,6} = 1/R$ and increase R until the system has the desired number of steady states.

4 From core to full models

In the previous sections we have analysed several systems based on a core model. That is, we have disregarded reverse reactions, hydrolysis reactions, and even complex formation in the phosphotransfer reactions. We provide here arguments that guarantee that the properties on the number of steady states of the different core models that we have considered extend to the full models.

4.1 Theoretical results

The results concerning the number of steady states extend to the full models. Two mathematical results, valid for mass-action kinetics, are used for this claim ([3], [4]):

[3] Assume that a network has N (nondegenerate) positive steady states. If complex formation is taken into account, that is, a reaction is split into two by adding an intermediate, then the new extended network also has N (nondegenerate) positive steady states for some choice of rate constants and total amounts.

[4] Assume that a network has N (nondegenerate) positive steady states. If reactions are added to the network, in such a way that the conservation laws of the system are preserved, then the new network also has N (nondegenerate) positive steady states for some choice of rate constants and total amounts.

The nondegeneracy condition means that the Jacobian is nonsingular relative to the stoichiometric compatibility class described by the conservation laws (cf. Subsection 2.4). This requirement is fulfilled in our case.

4.2 Full hybrid HK

The full model of a hybrid HK with reversible reactions and hydrolysis reactions, consists of the reactions:

$$\begin{array}{lll} \mathrm{HK_{00}} \xrightarrow{k_1} \mathrm{HK_{P0}} \xrightarrow{k_2} \mathrm{HK_{0P}} \xrightarrow{k_3} \mathrm{HK_{PP}} & & \mathrm{HK_{0P}} + \mathrm{Hpt} \underset{k_7}{\overset{k_4}{\rightleftarrows}} \mathrm{HK_{00}} + \mathrm{Hpt_P} \\ \mathrm{HK_{0P}} \xrightarrow{k_9} \mathrm{HK_{00}} & \mathrm{HK_{PP}} \xrightarrow{k_{10}} \mathrm{HK_{P0}} & \mathrm{HK_{PP}} + \mathrm{Hpt} \underset{k_8}{\overset{k_5}{\rightleftarrows}} \mathrm{HK_{P0}} + \mathrm{Hpt_P} \\ & \mathrm{RR_P} \xrightarrow{k_{13}} \mathrm{RR} & \mathrm{Hpt_P} + \mathrm{RR} \underset{k_{12}}{\overset{k_{11}}{\rightleftarrows}} \mathrm{Hpt} + \mathrm{RR_P}. \end{array} \tag{32}$$

One might also consider complex formation, that is, substitute each phosphotransfer reaction of the form $A + B \rightleftarrows C + D$ by

$$A + B \rightleftarrows Y \rightleftarrows C + D\ .$$

By Subsection 3.2, the core phosphorelay with a hybrid HK can have 3 positive steady states. Adding reversibility to some reactions does not change the conservation laws, nor does including hydrolysis reactions. Therefore, by [4], the full phosphorelay model with a hybrid HK can have 3 positive steady states for some choice of rate constants and total amounts. This holds true even if hydrolysis reactions are added on the other phosphorylation sites (that is, the first domain of the hybrid HK and Hpt). By [3], adding complex formation also maintains the maximal number of steady states. In both cases, however, a higher number of steady states might be achievable.

The same argument holds for all the models considered in the previous sections.

4.3 Unorthodox HK

Consider now the core model with unorthodox HK, obtained by removing some hydrolysis reactions and reversibility. HK has now three phosphorylable domains. The reactions of the system are as follows:

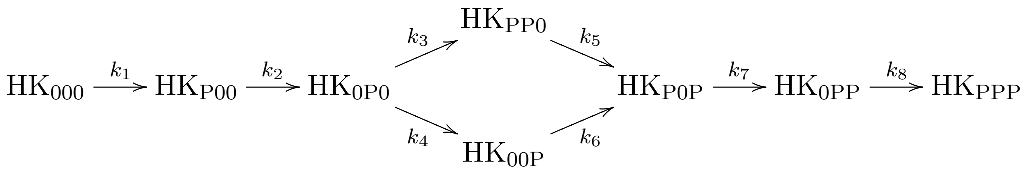

$$\mathrm{HK_{00P}} + \mathrm{RR} \xrightarrow{k_9} \mathrm{HK_{000}} + \mathrm{RR_P} \qquad \mathrm{HK_{P0P}} + \mathrm{RR} \xrightarrow{k_{10}} \mathrm{HK_{P00}} + \mathrm{RR_P}$$

$$\mathrm{HK_{0PP}} + \mathrm{RR} \xrightarrow{k_{11}} \mathrm{HK_{0P0}} + \mathrm{RR_P} \qquad \mathrm{HK_{PPP}} + \mathrm{RR} \xrightarrow{k_{12}} \mathrm{HK_{PP0}} + \mathrm{RR_P}$$

$$\mathrm{RR_P} \xrightarrow{k_{13}} \mathrm{RR}$$

We show here that this model is essentially obtained by adding iteratively species and reactions to the core hybrid HK model in Subsection 1.1, in the sense allowed by [4] and [3].

Not to confuse notation, we write the model in Subsection 1.1 with the different labels for the species:

$$A_1 \longrightarrow A_2 \longrightarrow A_3 \longrightarrow A_4$$

$$A_3 + B_1 \longrightarrow A_1 + B_2 \qquad A_4 + B_1 \longrightarrow A_2 + B_2 \qquad B_2 \longrightarrow B_1.$$

We perform the following modifications to the model:

(i) Add species C_1, C_2:

$$A_1 \longrightarrow C_1 \longrightarrow A_2 \longrightarrow A_3 \longrightarrow C_2 \longrightarrow A_4$$

$$A_3 + B_1 \longrightarrow A_1 + B_2 \qquad A_4 + B_1 \longrightarrow A_2 + B_2 \qquad B_2 \longrightarrow B_1.$$

(ii) Add a reaction from C_1 to A_3:

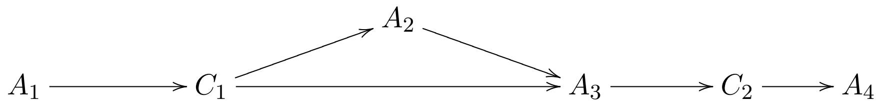

$$A_3 + B_1 \longrightarrow A_1 + B_2 \qquad A_4 + B_1 \longrightarrow A_2 + B_2 \qquad B_2 \longrightarrow B_1.$$

(iii) Add a species between C_1 and A_3:

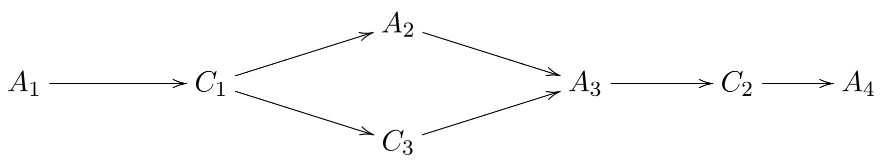

$$A_3 + B_1 \longrightarrow A_1 + B_2 \qquad A_4 + B_1 \longrightarrow A_2 + B_2 \qquad B_2 \longrightarrow B_1.$$

(iv) Add two reactions:

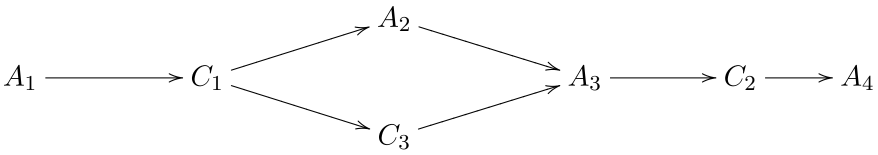

$$A_3 + B_1 \longrightarrow A_1 + B_2 \qquad A_4 + B_1 \longrightarrow A_2 + B_2 \qquad B_2 \longrightarrow B_1$$

$$C_3 + B_1 \longrightarrow A_1 + B_2 \qquad C_2 + B_1 \longrightarrow C_1 + B_2.$$

By either [4] or [3], each step preserves the number of positive steady states of the network of the previous step.

The network in (iv) is not exactly the core model of an unorthodox HK, but almost. The core model of the unorthodox HK requires an extra species C_5, a reaction $C_5 \to A_1$, and the reaction $C_3 + B_1 \to A_1 + B_2$ must be substituted by $C_3 + B_1 \to C_5 + B_2$. That is, the network we would like to have is

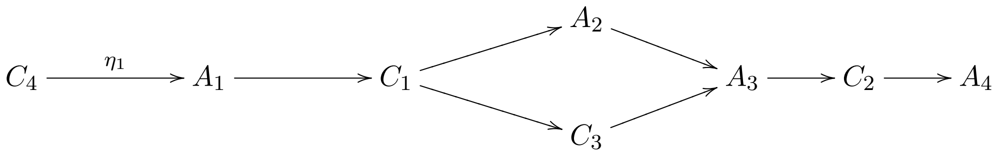

$$A_3 + B_1 \longrightarrow A_1 + B_2 \qquad A_4 + B_1 \longrightarrow A_2 + B_2 \qquad B_2 \longrightarrow B_1$$

$$C_3 + B_1 \xrightarrow{\eta_2} C_4 + B_2 \qquad C_2 + B_1 \longrightarrow C_1 + B_2.$$

The new species C_4 is not an intermediate, but behaves exactly like one. Consider the ODE system associated to this network. The steady state equation for $[C_4]$ gives at steady state

$$[C_4] = \frac{\eta_2}{\eta_1}[C_3][B_1].$$

Plugging this value back into the ODE system to eliminate $[C_4]$, we obtain the ODE system associated to the network in item (iv). This is the only characteristic of the intermediates that allows one to prove that the number of steady states is maintained. Therefore, the arguments given in [3] hold also for C_4.

We conclude that the core model of the unorthodox HK has at least 3 positive steady states for some choice of rate constants and total amounts, because this is the case for the core model for the hybrid HK. This has also been confirmed using the CRNT Toolbox [1].

This procedure applies as well to conclude that the system with n unorthodox HKs competing for the same RR can have $2n + 1$ positive steady states. Finally, the result holds as well if reactions are made reversible, hydrolysis reactions are added, or complex formation are considered.

5 Proof of the lemmas

5.1 Proof of Lemma 1

To simplify the notation, we prove that for all $\alpha_1, \ldots, \alpha_5 > 0$ there exist $k_1, \ldots, k_5, H > 0$ such that

$$\alpha_1 = k_1k_2k_4k_5H \qquad \alpha_2 = k_1k_2k_3k_5H \tag{33}$$

$$\alpha_3 = k_1k_4k_5 + k_2k_4k_5 \qquad \alpha_4 = k_1k_2k_5 + k_1k_3k_5 \qquad \alpha_5 = k_1k_2k_3. \tag{34}$$

Using (34) we solve iteratively for k_3, k_5 and k_4 and obtain:

$$k_3 = \frac{\alpha_5}{k_1k_2}, \qquad k_5 = \frac{\alpha_4k_2}{k_1k_2^2 + \alpha_5}, \qquad k_4 = \frac{\alpha_3(k_1k_2^2 + \alpha_5)}{\alpha_4k_2(k_1 + k_2)}. \tag{35}$$

Using the first equation in (33), we have

$$H = \frac{\alpha_2}{k_1k_2k_4k_5} = \frac{\alpha_2(k_1 + k_2)}{\alpha_3k_1k_2}. \tag{36}$$

Finally, using the second equation in (33), we have

$$0 = \alpha_2 - k_1k_2k_3k_5H = \alpha_2 - \frac{\alpha_2(k_1 + k_2)\alpha_4\alpha_5}{\alpha_3k_1(k_1k_2^2 + \alpha_5)},$$

which is equivalent to

$$0 = \alpha_2\alpha_3k_2^2k_1^2 + \alpha_2\alpha_5(\alpha_3 - \alpha_4)k_1 - \alpha_2\alpha_4\alpha_5k_2. \tag{37}$$

Fix any $k_2 > 0$. Since the polynomial (37) is a polynomial in k_1, with positive leading coefficient and negative independent term, it has a unique positive real root. As a consequence, for any $k_2 > 0$, (33) and (34) hold with k_1 defined such that (37) holds, and $k_3, k_4, k_5, H > 0$ as in (35), (36). □

5.2 Proof of Lemma 2

We have that

$$\begin{aligned} p(x) &= (x-1)\prod_{i=1}^{n}(x^2 + b_i) + \sum_{i=1}^{n}\Big(a_ix\prod_{j\neq i}(x^2 + b_j)\Big) \\ &= \prod_{i=1}^{n}(x^2 + b_i) + x\Big(\prod_{i=1}^{n}(x^2 + b_i) + \sum_{i=1}^{n}\big(a_i\prod_{j\neq i}(x^2 + b_j)\big)\Big). \end{aligned}$$

If we write

$$p_1(x) = \prod_{i=1}^{n}(x^2 + b_i) = \beta_0x^{2n} + \cdots + \beta_{2n-2}x^2 + \beta_{2n},$$

then a standard computation shows that

$$\beta_{2k} = \sum_{\{j_1,\ldots,j_k\}\subseteq[n]}\prod_{\ell=1}^{k} b_{j_\ell}, \qquad \text{and} \qquad \beta_{2k+1} = 0,$$

for $k = 0, 1, \dots, n$. In particular, only coefficients of even degree are nonzero. The second summand of $p(x)$,

$$p_2(x) = x\Big(\prod_{i=1}^{n}(x^2 + b_i) + \sum_{i=1}^{n}\big(a_i \prod_{j\neq i}(x^2 + b_j)\big)\Big),$$

has only terms of odd degree. Therefore, $c_{2k+1} = \alpha_{2k}$ as claimed and c_{2k} is the coefficient of degree $2n+1-2k$ of $p_2(x)$. Note that the polynomial $p_2(x)$ can be written as:

$$p_2(x) = xp_1(x) + x\sum_{i=1}^{n}\big(a_i \prod_{j\neq i}(x^2 + b_j)\big).$$

As above, if we write $\prod_{j\neq i}^{n}(x^2 + b_j) = \gamma_{i,0}x^{2n-2} + \cdots + \gamma_{i,2n-4}x^2 + \gamma_{i,2n-2}$, then

$$\gamma_{i,2k} = \sum_{\{j_1,\dots,j_k\}\subseteq[n]\setminus\{i\}} \prod_{\ell=1}^{k} b_{j_\ell}, \qquad \text{and} \qquad \gamma_{2k+1} = 0,$$

for $k = 0, 1, \dots, n-1$. The coefficient of degree $2n+1-2k$ of $p(x)$ is then

$$c_{2k} = \beta_{2k} + \sum_{i=1}^{n} a_i \gamma_{i,2k-2},$$

as claimed. □

5.3 Proof of Lemma 3

To simplify the presentation, we denote by $\mathcal{P}_k(n)$ the set of all subsets of $[n]$ with k elements. Similarly, given $i \in [n]$, we denote by $\mathcal{P}_{k-1}(n,i)$ the set of all subsets of $[n]$ with $k-1$ elements that do not contain i. We want to show that if we define $b_i = \frac{a_i^2}{4}$ for $i = 1, \dots, n$, then,

$$c_{2k}^2 - 4c_{2k-1}c_{2k+1} > 0$$

for all $k = 1, \dots, n$. Recall that we defined

$$c_{2k+1} = -\sum_{L\in\mathcal{P}_k(n)} \prod_{\ell\in L} b_\ell, \qquad c_{2k} = \sum_{L\in\mathcal{P}_k(n)} \prod_{\ell\in L} b_\ell + \sum_{i=1}^{n} a_i \sum_{J\in\mathcal{P}_{k-1}(n,i)} \prod_{j\in J} b_j.$$

It is clear that if $k \geq 1$, then

$$\begin{aligned} c_{2k}^2 &> \Big(\sum_{i=1}^{n} a_i \sum_{J\in\mathcal{P}_{k-1}(n,i)} \prod_{j\in J} b_j\Big)^2 \\ &> \sum_{i=1}^{n} \sum_{J\in\mathcal{P}_{k-1}(n,i)} a_i^2 \prod_{j\in J} b_j^2 + 2\sum_{i=1}^{n} \sum_{\substack{J,L\in\mathcal{P}_{k-1}(n,i) \\ J\neq L}} a_i^2 \prod_{j\in J} b_j \prod_{\ell\in L} b_\ell. \end{aligned} \tag{38}$$

On the other hand,

$$\begin{aligned}
4c_{2k+1}c_{2k-1} &= 4\left(\sum_{L\in\mathcal{P}_k(n)}\prod_{\ell\in L} b_\ell\right)\left(\sum_{J\in\mathcal{P}_{k-1}(n)}\prod_{j\in J} b_j\right) = 4\sum_{\substack{L\in\mathcal{P}_k(n)\\ J\in\mathcal{P}_{k-1}(n)}}\left(\prod_{\ell\in L} b_\ell \prod_{j\in J} b_j\right)\\
&= 4\left(\sum_{i=1}^{n}\sum_{J\in\mathcal{P}_{k-1}(n,i)} b_i\prod_{j\in J} b_j^2\right) + 4\sum_{\substack{L\in\mathcal{P}_k(n)\\ J\in\mathcal{P}_{k-1}(n), J\not\subseteq L}}\left(\prod_{\ell\in L} b_\ell\prod_{j\in J} b_j\right). \qquad (39)
\end{aligned}$$

If we set $b_i = \frac{a_i^2}{4}$, then the first two summands of (38) and (39) agree:

$$\sum_{i=1}^{n}\sum_{J\in\mathcal{P}_{k-1}(n,i)} a_i^2\prod_{j\in J} b_j^2 = 4\sum_{i=1}^{n}\sum_{J\in\mathcal{P}_{k-1}(n,i)}\frac{a_i^2}{4}\prod_{j\in J} b_j^2 = 4\left(\sum_{i=1}^{n}\sum_{J\in\mathcal{P}_{k-1}(n,i)} b_i\prod_{j\in J} b_j^2\right).$$

We let

$$\begin{aligned}
B_1 &= \sum_{i=1}^{n}\sum_{\substack{J,L\in\mathcal{P}_{k-1}(n,i)\\ J\neq L}} a_i^2\prod_{j\in J} b_j\prod_{\ell\in L} b_\ell = 4\sum_{i=1}^{n}\sum_{\substack{J,L\in\mathcal{P}_{k-1}(n,i)\\ J\neq L}} b_i\prod_{j\in J} b_j\prod_{\ell\in L} b_\ell\\
B_2 &= 2\sum_{\substack{L\in\mathcal{P}_k(n)\\ J\in\mathcal{P}_{k-1}(n), J\not\subseteq L}}\left(\prod_{\ell\in L} b_\ell\prod_{j\in J} b_j\right).
\end{aligned}$$

Then, to show that $c_{2k}^2 > 4c_{2k+1}c_{2k-1}$ it is enough to show that $\frac{B_1}{4} \geq \frac{B_2}{2}$. To this end, observe that given any pair of sets $L \in \mathcal{P}_k(n)$ and $J \in \mathcal{P}_{k-1}(n)$, such that $J \not\subseteq L$, we can choose $i \in L$ such that $L' := L \setminus \{i\}$ satisfies $L' \neq J$. Therefore, every summand of B_2 is a summand of B_1 as well. This finishes the proof. $\square$

5.4 Proof of Lemma 4

Let $\delta_k = c_{2k+1}^2 - 4c_{2k}c_{2k+2}$. For $M > 0$ and $a_1 > 0$, we let $a_i = \frac{a_n}{M^{i-1}}$ and $b_i = \frac{a_i^2}{4}$. Therefore, we have that

$$b_i = \frac{a_1^2}{4M^{2(i-1)}},$$

for $i = 1, \ldots, n$. With these substitutions, $\delta_k = \delta_k(a_1)$ becomes a polynomial in a_1. If there exists a choice of $M > 0$ such that the coefficient of *smallest degree* of δ_k is positive for all k, then $\delta_k(a_1) > 0$, for a_1 small enough and all k. Therefore, we compute the coefficient of smallest degree of δ_k in a_1.

We use the notation introduced in the proof of Lemma 3. By definition, we have that

$$\begin{aligned}
c_{2k+1}^2(a_1) &= \left(\sum_{J\in\mathcal{P}_k(n)}\prod_{j\in J}\frac{a_1^2}{4M^{2(j-1)}}\right)^2 = a_1^{4k}\left(\sum_{J\in\mathcal{P}_k(n)}\prod_{j\in J}\frac{1}{4M^{2(j-1)}}\right)^2\\
c_{2k}(a_1) &= \sum_{J\in\mathcal{P}_k(n)}\prod_{j\in J}\frac{a_1^2}{4M^{2(j-1)}} + \sum_{i=1}^{n}\frac{a_1}{M^{i-1}}\sum_{J\in\mathcal{P}_{k-1}(n,i)}\prod_{j\in J}\frac{a_1^2}{4M^{2(j-1)}}\\
&= a_1^{2k}\left(\sum_{J\in\mathcal{P}_k(n)}\prod_{j\in J}\frac{1}{4M^{2(j-1)}}\right) + a_1^{2k-1}\left(\sum_{i=1}^{n}\frac{1}{M^{i-1}}\sum_{J\in\mathcal{P}_{k-1}(n,i)}\prod_{j\in J}\frac{1}{4M^{2(j-1)}}\right)
\end{aligned}$$

The polynomial $c_{2k+1}^2(a_1)$ consists of one term of degree $4k$ in a_1. The polynomial $c_{2k}(a_1)$ is a sum of a term of degree $2k$ and one of degree $2k-1$. Similarly, the polynomial $c_{2k+2}(a_1)$ is a sum of a term of degree $2k+2$ and one of degree $2k+1$. Hence, the product $c_{2k}c_{2k+2}$ is a polynomial with lowest degree $4k$. If the coefficient of degree $4k$ of δ_k is nonzero, then it is the coefficient if smallest degree. By denoting the coefficient of degree $4k$ by β_k, we have:

$$\beta_k = \left(\sum_{J\in\mathcal{P}_k(n)} \prod_{j\in J} \frac{1}{4M^{2(j-1)}} \right)^2 - 4\left(\sum_{i=1}^{n} \frac{1}{M^{i-1}} \sum_{J\in\mathcal{P}_{k-1}(n,i)} \prod_{j\in J} \frac{1}{4M^{2(j-1)}} \right) \left(\sum_{s=1}^{n} \frac{1}{M^{s-1}} \sum_{L\in\mathcal{P}_{k+1}(n,s)} \prod_{\ell\in L} \frac{1}{4M^{2(\ell-1)}} \right).$$

For $M>1$, the largest summand in the positive summand of β_k is given by the choice of set $J=\{1,\dots,k\}\in\mathcal{P}_k(n)$ and takes the value

$$X_1 := \prod_{j=1}^{k} \frac{1}{16M^{4(j-1)}} = \frac{1}{4^{2k}M^{4\sum_{\ell=0}^{k-1}\ell}} = \frac{1}{4^{2k}M^{2k^2-2k}}.$$

The largest summand in the negative summand of β_k is given by the choice $i=k$, $s=k+2$, $J=\{1,\dots,k-1\}\in\mathcal{P}_{k-1}(n,i)$, and $L=\{1,\dots,k+1\}\in\mathcal{P}_{k+1}(n,s)$. Proceeding as above, the term takes the value

$$X_2 := 4\left(\frac{1}{M^{k-1}}\frac{1}{4^{k-1}M^{(k-1)(k-2)}}\right)\left(\frac{1}{M^{k+1}}\frac{1}{4^{k+1}M^{(k+1)k}}\right) = \frac{1}{4^{2k-1}M^{2k^2+2}}.$$

For M large enough, $X_1 > X_2$. It follows that for M large, X_1 dominates, and hence β_k is positive. This finishes the proof. □

References

[1] P. Ellison, M. Feinberg, H. Ji, and D. Knight. Chemical reaction network toolbox, version 2.2. Available online at `http://www.crnt.osu.edu/CRNTWin`, 2012.

[2] E. Feliu and C. Wiuf. Enzyme-sharing as a cause of multi-stationarity in signalling systems. *J. R. S. Interface*, 9(71):1224–32, 2012.

[3] E. Feliu and C. Wiuf. Simplifying biochemical models with intermediate species. *J. R. S. Interface*, 10:20130484, 2013.

[4] B. Joshi and A. Shiu. Atoms of multistationarity in chemical reaction networks. *J. Math. Chem.*, 51(1):153–178, 2013.

[5] D. C. Kurtz. A sufficient condition for all the roots of a polynomial to be real. *The American Mathematical Monthly*, 99:259–263, 1992.

[6] C. Wiuf and E. Feliu. Power-law kinetics and determinant criteria for the preclusion of multistationarity in networks of interacting species. *SIAM J. Appl. Dyn. Syst.*, 12:1685–1721, 2013.
